# TFEB controls sensitivity to chemotherapy and immuno-killing in non-small cell lung cancer

**DOI:** 10.1186/s13046-024-03142-4

**Published:** 2024-08-07

**Authors:** Muhlis Akman, Ciro Monteleone, Gabriella Doronzo, Martina Godel, Francesca Napoli, Alessandra Merlini, Virginia Campani, Valeria Nele, Elisa Balmas, Tatiana Chontorotzea, Simona Fontana, Sabrina Digiovanni, Francesca Alice Barbu, Elena Astanina, Niloufar Jafari, Iris Chiara Salaroglio, Joanna Kopecka, Giuseppe De Rosa, Thomas Mohr, Alessandro Bertero, Luisella Righi, Silvia Novello, Giorgio Vittorio Scagliotti, Federico Bussolino, Chiara Riganti

**Affiliations:** 1 Candiolo Cancer Institute, FPO-IRCCS, Candiolo, Turin, Italy; 2 IRCCS Candiolo Cancer Institute, Candiolo, Italy; 3 Pathology Unit, Department of Oncology at San Luigi Hospital, University of Torino, Torino, Italy; 4 Thoracic Oncology Unit, Department of Oncology at San Luigi Hospital, University of Torino, Torino, Italy; 5 Department of Pharmacy, University of Napoli Federico II, Napoli, Italy; 6 Department of Molecular Biotechnology and Health Sciences, University of Torino, Torino, Italy; 7 Center for Cancer Research and Comprehensive Cancer Center, Medical University Vienna, Vienna, Austria; 8 Molecular Biotechnology Center “Guido Tarone”, University of Torino, Torino, Italy

**Keywords:** TFEB, Chemo-immuno-resistance, ABCC1, ABCA1, Vγ9Vδ2 T-lymphocytes, Non-small cell lung cancer

## Abstract

**Background:**

In non-small cell lung cancer (NSCLC) the efficacy of chemo-immunotherapy is affected by the high expression of drug efflux transporters as ABCC1 and by the low expression of ABCA1, mediating the isopentenyl pyrophosphate (IPP)-dependent anti-tumor activation of Vγ9Vδ2 T-lymphocytes. In endothelial cells ABCA1 is a predicted target of the transcription factor EB (TFEB), but no data exists on the correlation between TFEB and ABC transporters involved in the chemo-immuno-resistance in NSCLC.

**Methods:**

The impact of TFEB/ABCC1/ABCA1 expression on NSCLC patients’ survival was analyzed in the TCGA-LUAD cohort and in a retrospective cohort of our institution. Human NSCLC cells silenced for TFEB (shTFEB) were analyzed for ABC transporter expression, chemosensitivity and immuno-killing. The chemo-immuno-sensitizing effects of nanoparticles encapsulating zoledronic acid (NZ) on shTFEB tumors and on tumor immune-microenvironment were evaluated in Hu-CD34^+^ mice by single-cell RNA-sequencing.

**Results:**

TFEB^low^ABCA1^low^ABCC1^high^ and TFEB^high^ABCA1^high^ABCC1^low^ NSCLC patients had the worst and the best prognosis, respectively, in the TCGA-LUAD cohort and in a retrospective cohort of patients receiving platinum-based chemotherapy or immunotherapy as first-line treatment. By silencing shTFEB in NSCLC cells, we demonstrated that TFEB was a transcriptional inducer of ABCA1 and a repressor of ABCC1. shTFEB cells had also a decreased activity of ERK1/2/SREBP2 axis, implying reduced synthesis and efflux via ABCA1 of cholesterol and its intermediate IPP. Moreover, TFEB silencing reduced cholesterol incorporation in mitochondria: this event increased the efficiency of OXPHOS and the fueling of ABCC1 by mitochondrial ATP. Accordingly, shTFEB cells were less immuno-killed by the Vγ9Vδ2 T-lymphocytes activated by IPP and more resistant to cisplatin. NZ, which increased IPP efflux but not OXPHOS and ATP production, sensitized shTFEB immuno-xenografts, by reducing intratumor proliferation and increasing apoptosis in response to cisplatin, and by increasing the variety of anti-tumor infiltrating cells (Vγ9Vδ2 T-lymphocytes, CD8^+^T-lymphocytes, NK cells).

**Conclusions:**

This work suggests that TFEB is a gatekeeper of the sensitivity to chemotherapy and immuno-killing in NSCLC, and that the TFEB^low^ABCA1^low^ABCC1^high^ phenotype can be predictive of poor response to chemotherapy and immunotherapy. By reshaping both cancer metabolism and tumor immune-microenvironment, zoledronic acid can re-sensitize TFEB^low^ NSCLCs, highly resistant to chemo- and immunotherapy.

**Supplementary Information:**

The online version contains supplementary material available at 10.1186/s13046-024-03142-4.

## Background

Lung cancer is the first cause of cancer-related death, with nearly 2 million deaths per year worldwide. The one-year survival rate is 50%, while the five-year survival rate drops to 19% [1]. Most of the newly diagnosed patients have a non-small cell lung cancer (NSCLC) histology, which can further be categorized as adenocarcinoma, squamous cell carcinoma and large cell carcinoma. The current treatment landscape is stage-dependent. Nowadays chemo-immunotherapy is considered the standard of care in advanced, unresectable disease, while in the last 5 years there have been a significant number of studies investigating the role of neo-adjuvant or perioperative chemo-immunotherapy in the setting of resectable disease [2, 3]. However, up to 30% of patients candidate to receive immunotherapy display resistance, making the quest for new agents effective against chemo-immuno-resistant NSCLC still an open challenge [4, 5].

Multidrug resistance is the major drawback of chemotherapy and is often associated with increased drug efflux via ATP-binding cassette (ABC) transporters, such as ABC subfamily B member 1/Multidrug resistance protein 1/P-glycoprotein (ABCB1/MDR1/Pgp) and ABC subfamily C member 1/Multidrug resistance-related protein 1 (ABCC1/MRP1) [6, 7]. ABCB1 and ABCC1 bind and hydrolyze ATP to transport endogenous metabolites and xenobiotics including chemotherapeutic drugs [8], negatively impacting on cancer treatment. By contrast, ABC subfamily A member 1 (ABCA1), known for its role in the efflux of intracellular cholesterol and assembly of nascent HDL, has a positive value. ABCA1 is responsible for the efflux of isopentenyl pyrophosphate (IPP), a small isoprenoid intermediate in cholesterol synthesis and an endogenous activator of the cytotoxic Vγ9Vδ2 T-lymphocytes [9]: through this mechanism, ABCA1 enhances tumor immuno-killing by this T-lymphocyte subset, while tumors with low ABCA1 are immuno-resistant [10, 11].

Besides the ABC-dependent mechanisms, drug resistance relies on a complex interplay of altered metabolism, altered organelle functions involving mitochondria metabolism and dynamics, endoplasmic reticulum and proteasome-dependent proteostasis, autophagy and endo-lysosomal functions [12–14]. Recently, a research line focused on the lysosomal compartment, which can sequester chemotherapeutic drugs that are weak bases, reducing their cytotoxic potential [15]. In this perspective, the transcription factor EB (TFEB), a leucine zipper protein belonging to the microphthalmia family of basic helix-loop-helix-leucine-zipper transcription factors (MiT family) that regulates lysosomal biogenesis and autophagy [16], has been proposed as a factor potentially coordinating drug resistance [15]. TFEB is retained in the cytosol as inactive form. Starvation, exercise and lysosomal stress lead to TFEB translocation into the nucleus, while the phosphorylation by extracellular signal-regulated kinase 1/2 (ERK1/2) and mammalian target of rapamycin complex 1 (mTORC1) prevents TFEB activity [17]. Among its pleiotropic functions, TFEB controls lipid and cholesterol homeostasis, by upregulating the scavenger receptor CD36 and stimulating lipophagy that fuels cells with fatty acids and cholesterol from lipid droplets [18, 19]. Furthermore, in breast cancer tumor-associated macrophages TFEB modulates anti-tumor immune response [20].

Currently there is no evidence of any correlation between TFEB, drug resistance or immuno-resistance mediated by ABC transporters. In previous studies on osteosarcoma and NSCLC, we demonstrated that ABCA1 and ABCB1/ABCC1 are reciprocally regulated [10, 11]. In NSCLC, the impaired mitochondrial oxidoreductive metabolism, which increases mitochondrial reactive oxygen species, activates the HIF-α/C/EBP-β axis that upregulates ABCC1 and downregulates ABCA1, producing chemo-immuno-resistance [10]. Interestingly, chromatin immunoprecipitation followed by sequencing (ChIP-seq; GSE88896) performed in TFEB^−/−^ human endothelial cells from umbilical cord veins indicated *ABCA1* as target of TFEB [21]. Since when ABCA1 is high, ABCC1 is low in NSCLC [10], and these two genes are significantly associated with patients’ prognosis according to The Cancer Genome Atlas (TCGA) analysis (https://portal.gdc.cancer.gov/projects/TCGA-LUAD), in this study we investigated if TFEB may control the levels of ABCA1 and ABCC1 in NSCLC. ABCB1 was excluded because it resulted less expressed in tumors than in non-tumor samples in TCGA-LUAD dataset, in contrast with other case series of NSCLC patients [22, 23]. We identified the molecular and metabolic circuitries linking TFEB with the expression and activity of ABCC1 and ABCA1 and controlling chemo- and immuno-sensitivity. By disrupting such circuitries, we set up a new pharmacological strategy that induces chemo-immuno-sensitization in NSCLC with low levels of TFEB, characterized by the worst response to chemotherapy and immuno-killing.

## Methods

### Chemicals and materials

Plasticware for cell culture was from Falcon (Becton Dickinson, Franklin Lakes, NJ). Fetal bovine serum (FBS) and culture medium were from Invitrogen Life Technologies (Carlsbad, CA). Electrophoretic reagents were from Bio-Rad Laboratories (Hercules, CA). BrHPP (IPH1101) was from MedKoo Biosciences Inc. (Morrisville, NC). If not otherwise specified, reagents were purchased from Sigma-Merck-Millipore (St. Louis, MO).

### TCGA analysis

Publicly available lung cancer datasets from TCGA were used and lung adenocarcinoma (LUAD) cases were selected (24; Supplemental Table S1). The transcriptome profiling of the TCGA-LUAD project was downloaded and imported to the R working environment (version 4.2.2). Count matrices and clinical information of TCGA-LUAD sequencing reads were extracted and exported. Count matrices were read, and Ensembl IDs of the genes were set. Feature annotations were created by querying “org.Hs.eg.db”, an annotation package based on mapping using Entrez Gene identifiers of human genes to obtain “ENSEMBL”, “ENTREZID”, “SYMBOL”, “GENENAME” features of the genes [25]. Counts matrices and feature annotations were synced to create a “dds” object, which was normalized via the DESeq2 package, to test differential expression by using negative binomial linear models, estimation of dispersion and logarithmic fold changes [26]. After creating a variance-stabilized transformed (VERSUST) dds object, the distributions of *TFEB*, *ABCA1* and *ABCC1* were compared in normal and tumor tissue. From these findings, “high” and “low” levels of the genes were estimated. Following gene dispersion, clinical data including the overall survival (OS) and the vital status of the cases were extracted, to correlate the co-expression of *TFEB*, *ABCA1* and *ABCC1* with OS. Weighted correlation network analysis (WGCNA), a R-package to find clusters (termed modules) of highly correlated genes, summarize these clusters using the “eigengene” function, was used to relate modules from one cluster to another [27]. The same dds object were filtered from microRNA, pseudogenes and uncharacterized genes, and loaded into variance stabilized transformation. Genes which have a base mean < 0 were filtered out. Filtered VERSUST objects were used to construct the network by the WGCNA package. Modules were set into biologic context by term enrichment analysis, the correlation between eigengene module and traits was calculated. Significantly differing eigengene modules were calculated by high versus low expression of the genes of interest, followed by the estimation of the connectivity and determination of hub genes.

### Retrospective analysis on NSCLC patients

A cohort of patients with unresectable stage III-IV NSCLC, treated with chemotherapy (cisplatin/carboplatin; *n* = 32) or immunotherapy (pembrolizumab; *n* = 43) as first-line treatment, was examined in terms of progression free survival (PFS: time from the beginning of treatment to the first sign of disease’s progression) and OS (time from the beginning of treatment until patients’ death) (Supplemental Table S2). RNA was extracted from each tumor sample and the levels of *TFEB*, *ABCA1* and *ABCC1* were measured by qRT-PCR, normalized on the housekeeping β-2-microglobulin gene (*B2M*). Patients were categorized in “high-” and “low-expressing” group, according to the median value of *TFEB*, *ABCC1* and *ABCA1*, and analyzed for PFS and OS. The study was conducted in accordance with the Declaration of Helsinki and was approved by the local ethics committee (San Luigi Gonzaga Hospital, Orbassano, Torino; IRB n. 73/2018).

### Cells

Calu-3, NCI-H1975, NCI-H3122, NCI-H2228, NCI-H441, NCI-H1650, A549 NSCLC cell lines were purchased from ATCC (Manassas, VA). Cells were maintained in RPMI-1640 (Gibco-Thermo Fisher Scientific, Waltham, MA) medium supplemented with 10% v/v FBS, 1% v/v penicillin-streptomycin, 1% v/v L-glutamine. Cell lines were authenticated by microsatellite analysis using a PowerPlex kit (Promega Corporation, Madison, WI; last authentication: September 2023). *Mycoplasma spp*. contamination was checked every 3 weeks using RT-PCR, and the contaminated cells were discharged.

### TFEB silencing and overexpression

A shRNA lentiviral vector produced in house was used to silence TFEB [21]. NCI-H441 and NCI-H2228 cells were seeded at 2.5 × 10^5^ cells/well. 24 h after seeding, cells were transduced with the vector targeting TFEB or with the corresponding empty vector (pLKO.1, Addgene, Watertown, MA) for 6 h, in medium containing 10 µg/mL polybrene/hexadimethrine bromide. For TFEB overexpression, 1.5 × 10^5^ cells were transduced for 6 h with a Tet-ON pLKO.1 vector containing the mutant, constitutively active TFEBS142A construct [28]. After incubation, the medium was removed and replaced by new medium with puromycin at the respective IC_50_ values (250 ng/mL for NCI-H441, 1 µg/mL for NCI-H2228). The efficiency of TFEB silencing or overexpression was checked with qRT-PCR at 24, 48 and 72 h. The best silencing conditions were: 48 h for NCI-H441 cells, 24 h for NCI-H2228 cells. To induce TFEB overexpression, 0.5 µg/mL doxycycline was added in the culture medium for 24 h.

### Flow cytometry

1 × 10^6^ cells were washed in phosphate-saline buffer (PBS), pH 7.2, 0.5% bovine serum albumin (BSA) and 2 mM EDTA, centrifuged at 300×g for 10 min, incubated 20 min at room temperature in the dark with 250 µL of Inside Fix reagent (Inside Stain Kit, Miltenyi Biotec., Bergisch Gladbach, Germany), centrifuged at 300×g for 5 min, washed with 1 mL of Inside Perm (Inside Stain Kit), centrifuged at 300×g for 5 min, and incubated 30 min at room temperature with the following antibodies (dilution 1/50): anti-MRP1/ABCC1 antibody (Miltenyi, Clone REA481, PE-conjugated); anti-ABCA1 (ThermoFisher, PA5-22908, DyLight 488-conjugated). Cells were washed with 1 mL of Inside Perm reagent, centrifuged at 300×g for 5 min and read using a Guava easyCyte Flow Cytometer (Millipore, Burlington, MA), equipped with the Guava Incyte software.

### qRT-PCR

Total RNA was extracted from cell lines and tumors, and reverse-transcribed using the iScript™ cDNA Synthesis Kit (Bio-Rad Laboratories). The qRT-PCR was performed with the IQ SYBR Green Supermix (Bio-Rad Laboratories). The list of primers is reported in Supplemental Table S3. The relative quantification of the genes of interest was performed by comparing each PCR product with the housekeeping *B2M* gene, using the Bio-Rad Software Gene Expression Quantitation (Bio-Rad Laboratories). In PCR arrays, 1 µg cDNA was loaded into “Lipoprotein signaling and cholesterol metabolism PrimePCR array” ready-to-use plates (Bio-Rad Laboratories).

### Immunoblotting

Cells were lysed in MLB buffer (125 mM Tris-HCl, 750 mM NaCl, 1% v/v NP40, 10% v/v glycerol, 50 mM MgCl_2_, 5 mM EDTA, 25 mM NaF, 1 mM NaVO_4_, 10 mg/mL leupeptin, 10 mg/mL pepstatin, 10 mg/mL aprotinin, 1 mM phenylmethylsulphonyl fluoride PMSF, pH 7.5), sonicated and centrifuged at 13,000×g for 10 min at 4 °C. Fifty µg of proteins were subjected to immunoblotting and probed with the following antibodies: anti-TFEB (Bethyl Laboratories, Inc., Montgomery, TX, A303-673 A, dilution 1/1000), anti-ABCA1 (Abcam, Cambridge, UK, clone HJI, dilution 1/500), anti-ABCC1 (Santa Cruz Biotechnology Inc., Santa Cruz, CA, sc-18835, dilution 1/1000), anti-phospho(Thr202/Tyr204)ERK1/2 (Cell Signalling Technology, Danvers, MA, clone AW39R, dilution 1/1000), anti-ERK1/2 (Cell Signaling Technology, 9102, dilution 1/1000), anti-glyceraldehyde 3-phosphate dehydrogenase (GAPDH; Santa Cruz Biotechnology Inc., sc-47724, dilution 1/1000). The proteins were detected by enhanced chemiluminescence (Bio-Rad Laboratories). In co-immunoprecipitation assays, 100 µg of whole cell lysates were immunoprecipitated with the following antibodies: anti-phospho(Thr202/Tyr204)ERK1/2 antibody (dilution 1/100), anti-sterol regulatory element binding protein 2 (SREBP2; NBP1-71880, Novus Biologicals, Littleton, CO, recognizing both uncleaved and cleaved SREBP2, dilution 1/500), anti-α-ubiquitin (R&D Systems, Minneapolis, MI, IgG_2B_ clone 83406, dilution 1/1000), anti-phospho-serine (Abcam, ab9332, dilution 1/1000), with the PureProteome Protein A/G Mix Magnetic Beads (LSKMAGAG10, Millipore) for 3 h at 4 °C. Immunoprecipitated samples were immunoblotted for SREBP2, phospho(Thr202/Tyr204)ERK1/2, ABCA1 and ABCC1. The band intensity was calculated with the Image J software, as ratio between density of protein of interest/density of housekeeping protein or band density between pERK1/2 and total ERK1/2. The band density of the protein of interest in each wild-type cell line was set as 1.

### Chromatin immunoprecipitation (ChIP)

5 × 10^6^ cells were resuspended with PBS containing 1% v/v formaldehyde. The cross-link reaction was stopped after 7 min by adding 125 mM glycine, then samples were centrifuged at 3,000×g for 1 min at 4^o^C. Pellets were resuspended in 1 mL chilled PBS containing 1 mM PMSF and 1X protease inhibitor cocktail III (Merck), then centrifuged twice at 3,000×g for 1 min at 4^o^C. The pellets were processed with the Zymo-Spin ChIP Kit (D5209-D5210, Zymo Research, Orange, CA), as per manufacturer’s instructions. ZymoMag Protein A beads were incubated with anti-TFEB antibody for 3 h at 4^o^C. Samples were then incubated at 75^o^C for 5 min with 5 M NaCl and centrifuged at 10,000×g for 30 s. The DNA eluted was incubated at 65^o^C for 30 min, then 1 µL Proteinase K was added for 90 min. The DNA was recovered with Zymo-Spin IC column with the DNA elution buffer of the kit. The promoter sequences were identified from the Eucaryotic Promoter Database (EPD) using *ABCA1* or *ABCC1* genes as inputs. The binding sites for TFEB were identified by Jaspar database [29]. The primers used for qRT-PCR after ChIP are reported in the Supplemental Table S3.

### Immunofluorescence microscope analysis

5 × 10^4^ wild-type and TFEB-silenced NCI-H2228 cells were seeded onto glass coverslips and incubated with the BioTracker™ 560 Orange Lysosome Dye (Merck), as per manufacturer’s instructions. Cells were fixed in 4% v/v paraformaldehyde, washed twice with PBS, incubated 1 h with an anti-ABCA1 (PA1-16789; Invitrogen) or an anti-ABCC1 (PA5-114802, Invitrogen) antibody, washed with PBS and incubated 30 min at room temperature with the anti-rabbit antibody DyLight^™^ 488 (SA5-10110, Invitrogen) or the anti-mouse antibody Alexa Fluor488 (A-10631, Invitrogen) antibodies. Samples were washed 5-times with PBS and counterstained with 4’,6-diamidino-2-phenylindole (DAPI; Merck, diluted 1/10000) for 3 min at room temperature in the dark. After 3 washes in PBS and 1 wash in deionized H_2_O, the slides were mounted with 4 µL of Gel Mount Aqueous Mounting (Merck) and examined by a Leica DC100 fluorescence microscope (Leica Microsystem, Wetzlar, Germany).

### ERK activity

MAP Kinase Assay Kit (17–191, Millipore) was used to measure ERK1/2 activity. Two hundred µg of cell lysates were immunoprecipitated with anti-ERK1/2 (137F5, Cell Signaling Technology, dilution 1/1000) antibody, then Mg^2+^/ATP cocktail and the kinase substrate from the kit were added for 30 min, in the absence or presence of the ERK inhibitor of the kit. After this incubation time, 1 µg proteins were subjected to immunoblotting and probed with the anti-phospho-ERK1/2 antibody supplied by the kit (dilution 1/1000). Band intensity, calculated with Image J software, was considered an index of ERK1/2 activity.

### Synthesis and efflux of cholesterol and IPP

Cells were labeled with 1 µCi of [^3^H]-acetate (3600 mCi/mmol; Amersham International, Piscataway, NJ) for 24 h. The synthesis of radiolabeled cholesterol and IPP was measured after lipid extraction, separation by thin layer chromatography (TLC) and liquid scintillation count [9]. Results were expressed as fmoles/mg cell proteins, according to the relative calibration curves. The efflux of an exogenous pulse of cholesterol and IPP was measured by radiolabeling cells for 1 h with [^3^H]-acetate (3600 mCi/mmol; Amersham International) or [^14^C]-IPP (50 mCi/mmol; Amersham International), washing five times with PBS and letting cells for 24 h in fresh medium. After this incubation time, lipids were extracted from supernatants and separated by TLC. Cholesterol and IPP were quantified by liquid scintillation [9]. Results were expressed as pmoles/mL, according to the relative calibration curves.

### Vγ9Vδ2 T-lymphocyte activation and immuno-killing

Peripheral blood samples were obtained from healthy blood donors (AOU Città della Salute e della Scienza, Torino, Italy; DG 767/2015). After isolation on a Ficoll-Hypaque density gradient, peripheral blood mononuclear cells (PBMC) were subjected to an immuno-magnetic sorting with the TCRγ/δ^+^T Cell Isolation Kit (Miltenyi Biotec.) [30]. The phenotypic characterization of Vγ9Vδ2 T-lymphocytes was confirmed by staining 5 × 10^5^ isolated cells with anti-TCR Vγ9 (REA470, FITC-conjugated), anti-Ki67 (REA183, PE-conjugated) and anti-INF-γ (REA600, APC-conjugated) antibodies (Miltenyi Biotec., diluted 1/10). Cells were counted with a BD Accuri™ C6 Plus Flow Cytometer. Results were expressed as percentage of Vγ9^+^/Ki67^+^/IFNγ^+^cells over Vγ9^+^cells. To measure the Vγ9Vδ2 T-lymphocyte-mediated immuno-killing, 5 × 10^5^ Vγ9Vδ2 T-lymphocytes were cultured overnight with NSCLC cells at 1:1 ratio, then NSCLC cells were washed twice with PBS, detached with Cell Dissociation Solution and stained with the Annexin V/Propidium Iodide kit (APOAF, Sigma-Merck), as per manufacturer’s instruction. The fluorescence was acquired with a BD Accuri™ C6 Plus Flow Cytometer. The results were expressed as killing fold change, i.e., percentage of Annexin V^+^/Propidium Iodide^+^ cells in each experimental condition/percentage of Annexin V^+^/Propidium Iodide^+^ untreated cells [10].

### Total and mitochondrial cholesterol

To measure total cholesterol, 10 × 10^6^ cells were lysed in 0.5 mL of 10 mM Tris, 100 mM NaCl, 20 mM KH_2_PO_4_, 30 mM EDTA, 1 mM EGTA, 250 mM sucrose, pH 7.5, and sonicated with 2 bursts of 10 s (Labsonic sonicator, Sartorius Stedim Biotech S.A., Aubagne Cedex, France), then centrifuged at 13,000×g for 15 min at 4 °C. The supernatants were centrifuged at 100,000×g for 1 h at 4 °C, using an Optima L-90 K Beckman Coulter Ultracentrifuge (Beckman Coulter Inc, Fullerton, CA) to collect the membrane fractions. For mitochondrial cholesterol, the mitochondrial extracts were prepared as previously described [10]. The pellets of membranes or mitochondria were resuspended in 250 µL of the assay buffer provided by the fluorometric Cholesterol/Cholesteryl Ester Assay Quantitation kit (ab65359, Abcam), to measure free cholesterol in the membrane as per manufacturer’s instructions, with a Synergy HT Multi-Detection Microplate Reader (Bio-Tek Instruments, Winooski, VT). An aliquot of 50 µL was sonicated and used to measure the proteins. Results were expressed as nmoles cholesterol/mg membrane or mitochondrial proteins.

### Electron transport chain (ETC) activity, O2 consumption rate (OCR) and mitochondrial ATP

The electron efflux from complex I to complex III, taken as an index of the mitochondrial respiratory activity, was assessed by measuring the rate of cytochrome c reduction in isolated mitochondria, reading the absorbance changes at 550 nm by a Synergy HT Multi-Detection Microplate Reader (Bio-Tek Instruments) [10]. Results were expressed as nanomoles of reduced cytochrome c/min/mg mitochondrial proteins. For OCR, 5 × 10^4^ NCI-H441 and NCI-H2228 cells were seeded in 96-well microplates (Nunc, Rochester, NY). After 24 h, the Resipher oxygen sensing lid (Lucid Scientific, Atlanta, MA) was positioned upon the plate. Cells were incubated with cisplatin at respective IC_25_-IC_50_-IC_75_ and monitored continuously for 130 h by measuring the flux of O_2_ diffusing into the cells from the air above the well. Data were analyzed using the Resipher web application (Lucid Scientific) [10]. Mitochondrial ATP was measured with the ATP Bioluminescent Assay Kit (FLAA; Sigma Aldrich), as per manufacturer’s instructions. Results were expressed as nanomoles/mg mitochondrial proteins.

### ABCC1 activity

Plasma-membrane vesicles enriched of ABCC1 were prepared by sequential centrifugation as detailed previously [31]. One hundred µg proteins were immunoprecipitated in non-denaturing conditions using an anti-ABCC1 (ab263865, Abcam, dilution 1/100) antibody, in the presence of 25 µL of PureProteome Protein A/G Mix Magnetic Beads. The ATPase activity of immunopurified ABCC1 was evaluated spectrophotometrically by measuring the absorbance of the phosphate hydrolyzed from ATP at 620 nm, using a Synergy HT Multi-Detection Microplate Reader (Bio-Tek Instruments). The absorbance was converted into µmoles of hydrolyzed phosphate/min/mg proteins, according to the titration curve previously prepared.

### [^3^H-carboplatin] accumulation

Cells were incubated for 3 h with 1 µCi/mL [^14^C]-carboplatin (20 Ci/mmol; Amersham Bioscience), washed twice in PBS, detached with trypsin, centrifuged at 1300×g for 2 min and sonicated. The amount of [^14^C]-carboplatin was quantified by liquid scintillation. Radioactivity was converted in nmoles/mg cell proteins.

### Cell viability

Cell viability against increasing concentrations of cis-diammine platinum (Il) dichlorate (cisplatin) and paclitaxel was measured with the WST-1 kit (Roche, Basel, Switzerland) as per manufacturer’s instructions, using a Synergy HT Multi-Detection Microplate Reader (Bio-Tek Instruments). The relative absorbance units of untreated cells was considered as 100% viability; the results were expressed as a percentage of viable cells versus untreated cells.

### Self-assembled zoledronic acid nanoparticles

Self-assembling nanoparticles encapsulating zoledronic acid (termed NZ) were prepared and characterized as previously reported [32].

### In vivo experiments

1 × 10^6^ NCI-H2228 wild-type (WT), TFEB-silenced (shTFEB) cells and shTFEB cells overexpressing TFEBS142A (ovTFEB), mixed with 100 µL Matrigel (Merck), were injected subcutaneously in female NOD SCID-γ (NSG) mice or in NSG mice engrafted with human hematopoietic CD34^+^ cells (Hu-CD34^+^; The Jackson Laboratories, Bar Harbor, MA). Mice were housed (*n* = 5 per cage) under 12 h light/dark cycle, with food and drinking provided ad libitum. To maintain the overexpression of TFEB, 1 mg/mL doxycycline was added daily to the drinking water [33] of the mice bearing ovTFEB tumors. The levels of TFEB were verified in the explanted tumors by RT-PCR. Tumor growth was measured weekly by caliper, according to the equation (LxW2)/2, where L = tumor length and W = tumor width. When tumors reached the volume of 50 mm^3^, animals (*n* = 5/group) were randomized. In a first experimental set, mice bearing WT, shTFEB and ovTFEB tumors were treated for 3 weeks as it follows: (1) control group, treated with 0.1 mL saline solution intravenously (i.v.), once a week; (2) cisplatin group, treated with 2 mg/kg cisplatin i.v., once a week. In a second experimental set, mice bearing WT and shTFEB tumors were treated for 3 weeks as it follows: (1) control group, treated with 0.1 mL saline solution i.v., once a week; (2) cisplatin group, treated with 2 mg/kg cisplatin i.v., once a week; (3) NZ, treated with 1 mg/kg NZ i.v., once a week; (4) NZ + cisplatin group, receiving both drugs i.v., once a week simultaneously. Animals were euthanized on day 28 after randomization with zolazepam (0.2 mL/kg) and xylazine (16 mg/kg). Animal weights were monitored throughout the study. Tumors were excised, weighted, and photographed. Tumor sections, fixed in 4% v/v paraformaldehyde, were stained with hematoxylin/eosin and anti-Ki67 antibody (Merck, dilution 1/100) followed by a peroxidase-conjugated secondary antibody (Dako, Santa Clara, CA, dilution 1/1000). Nuclei were counterstained with hematoxylin. Tumor tissues were also stained with in situ Cell Death Detection Kit (TUNEL Assay; Roche), followed by nuclei counterstaining with DAPI. Sections were examined with a LeicaDC100 microscope. Immunostaining quantification was performed using the ImageJ software: the results were expressed as percentage of Ki67- or TUNEL-positive nuclei over 100 nuclei counted. In each sample 5 fields were analyzed. Immediately after the euthanasia, 200 µL blood were collected to measure the following hematochemical parameters: red blood cells (RBC), white blood cells (WBC), hemoglobin (Hb), platelets (PLT), as indexes of bone marrow function; lactate dehydrogenase (LDH), aspartate aminotransferase (AST), alanine aminotransferase (ALT), alkaline phosphatase (AP), as indexes of liver function; creatinine, as index of kidney function; creatine phosphokinase (CPK), as index of muscle/heart damage, using commercially available kits from Beckman Coulter Inc. Heart, lungs, liver, kidneys and spleen were collected and fixed in 4% v/v paraformaldehyde. The sections were stained with hematoxylin-eosin (Sigma Aldrich) and examined with a LeicaDC100 microscope. Animal care and experimental procedures were approved by the Italian Ministry of Health (#627/2018-PR, 10/08/2018).

### Single cell RNA-sequencing (scRNA-Seq) analysis

A total of 20 freshly isolated samples were analyzed using microfluidic-based scRNA-Seq in 4 batches. Excised tumor tissues were cleared from fat, fibrous and necrotic areas, cut into ~ 1 mm^3^ pieces, and dissociated using the gentleMACS Tissue Dissociator kit (130-095-929, Miltenyi Biotec.) and the Cell Debris Removal kit (130-109-398, Miltenyi Biotec.). 5 mL of enzyme mix (4.675 mL RPMI + 200 µL Enzyme H + 100 µL Enzyme R + 25 µL Enzyme A) per tumor sized 0.2–1.0 g was added to gentleMACS™ C Tube and incubated in the gentleMACS Dissociator for 1 h. Cell suspension was filtered through a 70 µm-strainer and centrifuged at 300×g for 7 min. Supernatants were removed and suspensions were diluted with Red Blood Cell Lysis Solution (130-094-183, Miltenyi Biotec.). After cold PBS was overlaid, samples were centrifuged at 3000×g for 10 min at 4°C. The top-2 phases were aspirated, and cold PBS was added. Tubes were inverted gently three times and centrifuged at 3000×g for 10 min at 4°C. After discarding supernatants, cells were resuspended with 1 mL PBS/0.04% w/v BSA and live cells were counted using a Countless II device (Invitrogen). For each sample, 1 × 10^6^ live cells were transferred to 2 mL DNA LoBind tube (Eppendorf, Hamburg, Germany) in a total volume of 1 mL PBS/0.04% w/v BSA and centrifuged at room temperature at 300×g for 5 min. After removing supernatant, each pellet was resuspended in 100 µL of a distinct Cell Multiplexing Oligo (CMO; 3’ CellPlex Kit Set A, PN-1000261, 10X Genomics; Pleasanton, CA). After 5 min of incubation at room temperature, cells were transferred on ice, washed 3 times with 2 mL ice-cold PBS/1% w/v BSA by centrifuging at 300×g for 5 min at 4 °C. After the last wash, cells were incubated with 10% v/v 7-aminoactinomycin D (7-AAD) in 200 µL PBS/2% w/v BSA for 10 min. 5 × 10^5^ 7-AAD^+^(live) single cells per sample were sorted with Sony SH800S Cell Sorter (Sony Biotechnology, San Jose, CA) in sterile tubes kept at 4 °C, containing 100 µL RPMI + 0.2 U/mL RNAse inhibitor (ThermoFisher Scientific). Samples were subsequently pooled in a 2 mL DNA LoBind tube, pelleted at 300×g for 5 min at 4 °C, resuspended in 200 µL and diluted to a final concentration of 1.5 × 10^6^ cells/mL. 49,500 cells were loaded in a single channel of a Chromium Next GEM Chip G to prepare scRNA-Seq libraries using the Chromium Next GEM Single Cell 3ʹ Reagent Kit v3.1 and the Chromium X controller (all from 10X Genomics), following the manufacturer’s instructions to prepare gene expression (GEX) and CMO libraries (protocol CG000388 Rev B). All GEMs from the 4 batches were processed together for library preparation. GEMs were broken and cDNAs were purified, pre-amplified by 11 cycles of PCR, and quality controlled using an Agilent D5000 ScreenTape and an Agilent 2150 Tapestation (Agilent, Santa Clara, CA). 25% of each cDNA was fragmented, end-repaired, A-tailed, ligated to Illumina adapters, and amplified by 12 cycles of PCR using an individual primer pair from the Dual Index Kit TT, Set A (10X Genomics) to generate GEX libraries. CMO libraries were generated using the Dual Index Kit NN, Set A (10X Genomics), quality controlled using an Agilent D1000 ScreenTape and an Agilent 2150 Tapestation, quantified using the Qubit dsDNA HS Assay (Invitrogen), and pooled at 6:1 molar ratio for GEX:CMO. The library pool was sequenced using an Illumina NovaSeq 6000 and an Illumina S4 200 (Illumina, San Diego, CA) flow cell, performing 28 cycles for read 1, 90 cycles for read 20, and 10 cycles for each index. Raw 10x reads were demultiplexed into fastq files for individual libraries using Cellranger (version 7.0.0), which was also used to demultiplex samples based on CMOs, align unique reads to map the reads to the reference genome, assign reads to individual cells based on 10X Genomics barcodes, remove duplicate reads and filter for valid cells. Quality control was done by examining the number of reads per cell, the read distribution and the percentage of reads mapped to the mitochondrial genome. Data normalization and VERSUST were performed to preprocess the data. The Seurat package (version 4) was used to filter out cells with low gene expression or high mitochondrial gene expression, and to analyze counts matrices [34]. The gene.versus.molecule.cell.filter function from the Pagoda2 package was used to exclude cells with anomalously high gene counts or sizes. The PercentageFeatureSet function of the Seurat package was used to calculate the proportion of mitochondrial reads and discard samples when over 5% of the counts mapped the mitochondrial genes [35]. FindVariableFeatures function was used to identify highly variable genes and principal component analysis (PCA) was performed on these genes to reduce the dimensionality of the data. RunUMAP (Uniform Manifold Approximation and Projection) function was applied to visualize high-dimensional data in two or three dimensions. FindIntegrationAnchors function was used to identify and remove batch effects. UMAP embeddings were generated using the top 30 principal components with a resolution of 0.5 for clustering and differential expression analysis. To standardize data, ScaleData function was used. Louvain algorithm was used to cluster cells with the goal of optimizing the standard modularity function FindClusters resolution 0.5. FindMarkers function was applied to identify differentially expressed genes between cluster of cells, using a non-parametric Wilcoxon rank-sum test to compare the gene expression levels between two groups of cells. The resulting p values were adjusted for multiple testing using Bonferroni correction [36]. For gene set enrichment analysis (GSEA), scRNA Seq data were converted to static files by using “pagoda2” package [37]. These files were loaded to pagoda2 frontend web application to find tumor cells in the clusters [38]. Clusters were selected and subjected to differential expression analysis provided by the web application to obtain the genetic profile of each cluster and identify tumor cells. Results were saved as .csv files. Differentially expression results were then processed by using “clusterprofiler” package [39]. The biologic process section of Gene ontology database [40] was used to identify the pathways related to genes obtained during over-representation analyses. Results were represented as dot-plots and network plots.

### Statistical analysis

All data in the text and figures are provided as means ± SD. The results were analyzed by a two-way analysis of variance (ANOVA), using GraphPad Prism 9 (Dotmatics, version 9.5.1). *p* < 0.05 was considered significant. Pearson correlation coefficients were calculated based on fold-changes of TFEB, ABCA1, and ABCC1 mRNA levels, then the matrix was created based on coefficients ranging from − 1 to + 1, where − 1 means negative correlation while + 1 means perfect correlation. Kaplan-Meier survival analysis was performed to calculate the PFS and OS. Log-rank test was used to compare the outcome of TFEB^low^ABCA1^low^ABCC1^high^ and TFEB^high^ABCA1^high^ABCC1^low^ patients. To adjust p-value, Benjamini & Hochberg (BH) method was used in over-representation and GSEA.

## Results

### Co-expression patterns of TFEB and ABC transporters predict survival in NSCLC patients

**Fig. 1:**
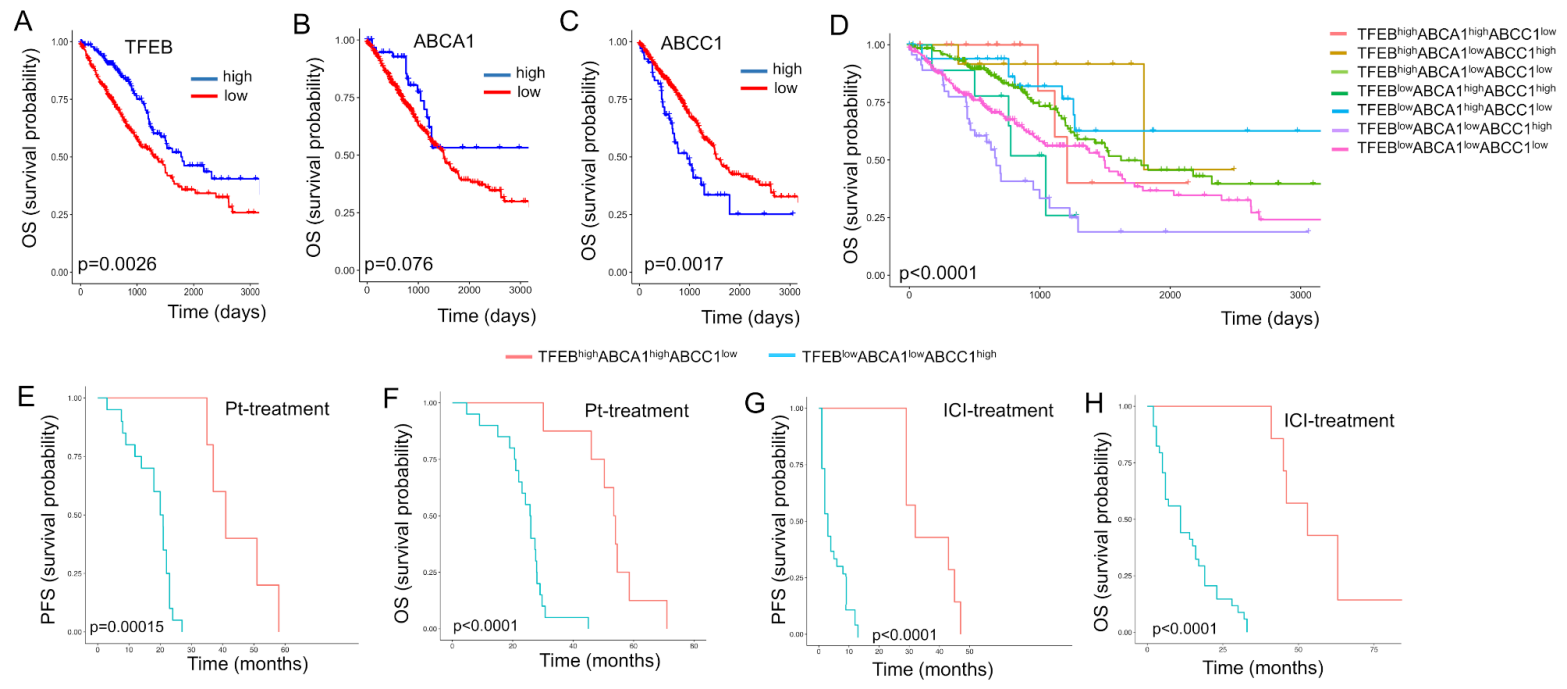
Impact of the expression of TFEB, ABCA1 and ABCC1 on NSCLC patients’ survival. (**A-C**) Kaplan Meyer analysis of overall survival (OS) of patients from the TGCA-LUAD cohort (*n* = 531), categorized according to the expression levels of TFEB (**A**), ABCA1 (**B**) and ABCC1 (**C**). (**D**) OS in TGCA-LUAD cohort patients, categorized in: TFEB^high^ABCA1^high^ABCC1^low^, TFEB^high^ABCA1^low^ABCC1^high^, TFEB^high^ABCA1^low^ABCC1^low^, TFEB^low^ABCA1^high^ABCC1^high^, TFEB^low^ABCA1^high^ABCC1^low^, TFEB^low^ABCA1^low^ABCC1^high^, TFEB^low^ABCA1^low^ABCC1^low^. (**E-H**) Retrospective analysis of progression-free survival (PFS; **E**, **G**) and OS (**F**, **H**) in TFEB^high^ABCA1^high^ABCC1^low^ versus TFEB^low^ABCA1^low^ABCC1^high^ patients, treated with cisplatin/carboplatin (Pt; *n* = 32) or pembrolizumab as immune checkpoint inhibitor (ICI; *n* = 43) at the Department of Oncology, University of Torino, Italy

We analyzed the TCGA-LUAD dataset (*n* = 585): after excluding 8 cases because of the lack of information about tumor stage and 59 cases classified as “normal tissues”, we analyzed the expression of TFEB, ABCC1 and ABCA1 mRNA in the remaining cohort of 531 cases of primary tumors (Supplemental Table S1). High TFEB expression significantly predicted a better OS (Fig. 1A). A similar trend, although not significant, was observed for the immuno-sensitizing gene ABCA1 (Fig. 1B), while high levels of ABCC1 were associated with poor OS (Fig. 1C). These results were validated in an internal cohort of patients with advanced NSCLC, treated with cisplatin/carboplatin (*n* = 32) or pembrolizumab (*n* = 43) as first line (Supplemental Table S2): in both chemotherapy or immunotherapy-treated patients, high TFEB and ABCA1 were associated with better PFS and OS, high ABCC1 with worse outcome (Supplemental Fig. S1A-B). In a subsequent co-expression analysis, we found that the TFEB^low^ABCA1^low^ABCC1^high^ phenotype was the poorest in terms of survival amongst all phenotypes, while the TFEB^high^ABCA1^high^ABCC1^low^ phenotype was the best, in TCGA-LUAD cohort (Fig. 1D) and in the retrospective in-house analysis of patients treated with chemotherapy (Fig. 1E-F) or immunotherapy (Fig. 1G-H).

### TFEB up-regulates ABCA1 and down-regulates ABCC1 in non-small cell cancer cells

**Fig. 2:**
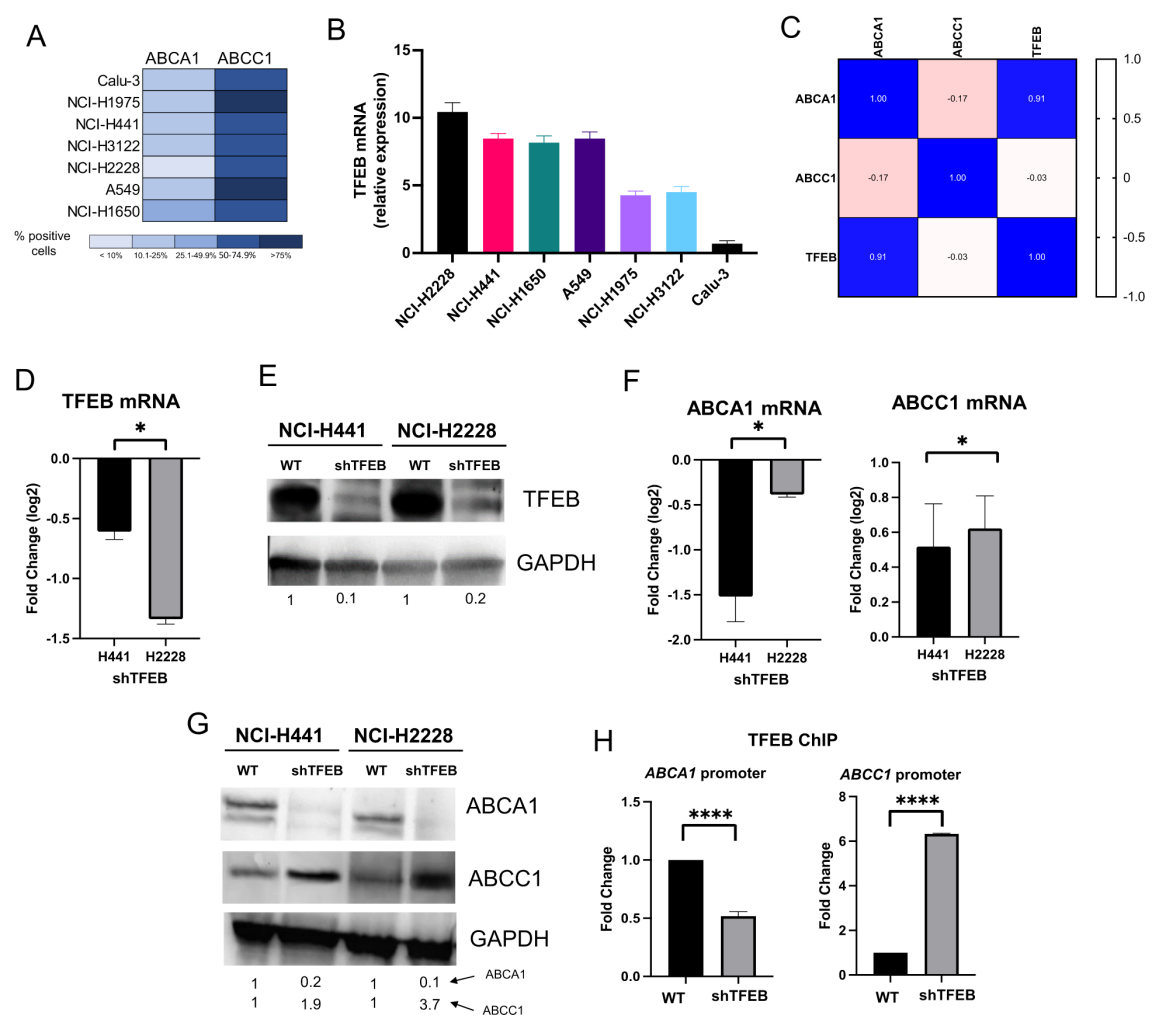
Changes in the expression levels of TFEB, ABCA1 and ABCC1 in NSCLC cell lines. (**A**) Surface amount of ABCA1 and ABCC1, measured by flow cytometry in duplicates (*n* = 3 independent experiments), in 6 NSCLC cell lines. Results are presented as a heatmap using a colorimetric scale, indicating the percentage of positive cells for ABCA1 or ABCC1. (**B**) TFEB mRNA measured by qRT-PCR in triplicates. Data are means *±* SD (*n* = 3 independent experiments). (**C**) Correlation matrix between the expression levels of TFEB, ABCA1 and ABCC1, based on the results of panels A-B. (**D**). TFEB mRNA in wild-type (WT) NCI-H441 and NCI-H2228 cells transduced with the pLKO empty vector and in cell lines silenced for TFEB (shTFEB), measured by RT-PCR in triplicates. Data are means ± SD of the mRNA fold change (*n* = 3 independent experiments). *p < 0.05: shTFEB versus WT cells. (**E**) Immunoblot of TFEB protein in WT and shTFEB cells. GAPDH was used as control of equal protein loading (*n* = 3 independent experiments). The band density of TFEB is indicated below the image. (**F**) ABCA1 and ABCC1 mRNA in shTFEB cells (versus WT cells), measured by RT-PCR in triplicates. Data are means ± SD of the mRNA fold change (*n* = 3 independent experiments). *p < 0.05: shTFEB versus WT cells. (**G**) Immunoblot of ABCA1 and ABCC1. GAPDH was used as control of equal protein loading (*n* = 3 independent experiments). The band density of ABCA1 and ABCC1 is indicated below the image. (**H**) TFEB binding to *ABCA1* and *ABCC1* promoter in WT and shTFEB NCI-H2228 cells, evaluated by ChIP followed by RT-PCR. Data are means ± SD (*n* = 3 independent experiments). ****p < 0.0001: shTFEB versus WT cells

Afterward, we analyzed the expression of the TFEB, ABCA1 and ABCC1 in a panel of 6 NSCLC cell lines, with a different degree of resistance to cisplatin and to Vγ9Vδ2 T-lymphocyte immuno-killing [15]. Notwithstanding the intercellular differences, all the NSCLC cells had < 50% cells positive for ABCA1, > 50% cells positive for ABCC1 (Fig. 2A). TFEB mRNA was detected in all cell lines (Fig. 2B): interestingly, it was positively correlated with ABCA1, negatively correlated with ABCC1 in the pool of cell lines analyzed (Fig. 2C). The TFEB highly expressing NCI-H2228 and NCI-H441 cells were chosen for the following experiments. In these cell lines silenced for TFEB (Fig. 2D-E), ABCA1 was decreased and ABCC1 was increased at both mRNA (Fig. 2F) and protein level (Fig. 2G). We hypothesized that the effect of TFEB on ABC transporters may be at transcriptional level, since previous data of ChIP-seq in endothelial cells reported *ABCA1* as a target gene of TFEB [21]. Putative binding sites for TFEB were present in both promoters. ChIP assays indicated that shTFEB cells had lower and higher transcriptional activity of *ABCA1* and *ABCC1* promoters, respectively (Fig. 2H), indicating that TFEB up-regulates ABCA1 and down-regulates ABCC1.

Post-translation events as ubiquitination or phosphorylation of ABC transporters may decrease or increase chemoresistance, respectively [41]. However, TFEB-silenced cells had no changes in ABCA1 and ABCC1 ubiquitination or phosphorylation (Supplemental Fig. S2A-D). Similarly, an accelerated endocytosis of ABC transporters via endosomal/lysosomal system followed by their degradation determines chemosensitivity [42]. As expected, given the central role of TFEB in promoting lysosome biogenesis [16], wild-type NCI-H2228 cells had more lysosomes than TFEB-silenced counterpart. In immunofluorescence assays, ABCA1 and ABCC1 staining resulted more intense in wild-type and shTFEB cells, respectively, but none of the transporters co-localized with lysosomes (Supplemental Fig. S3A). Also, ATP7B, a lysosomal protein that is induced by TFEB and mediates the cisplatin lysosomal sequestration in resistant ovarian cancer cells [43], was down-regulated in TFEB-silenced cells compared to wild-type cells (Supplemental Fig. S3B), excluding that it was involved in cisplatin resistance upon TFEB silencing.

### TFEB silencing impairs cholesterol homeostasis by inhibiting ERK1/2/SREBP2 axis and ABCA1/IPP/Vγ9Vδ2 T-cell-mediated immuno-killing of non-small cell lung cancer cells

**Fig. 3:**
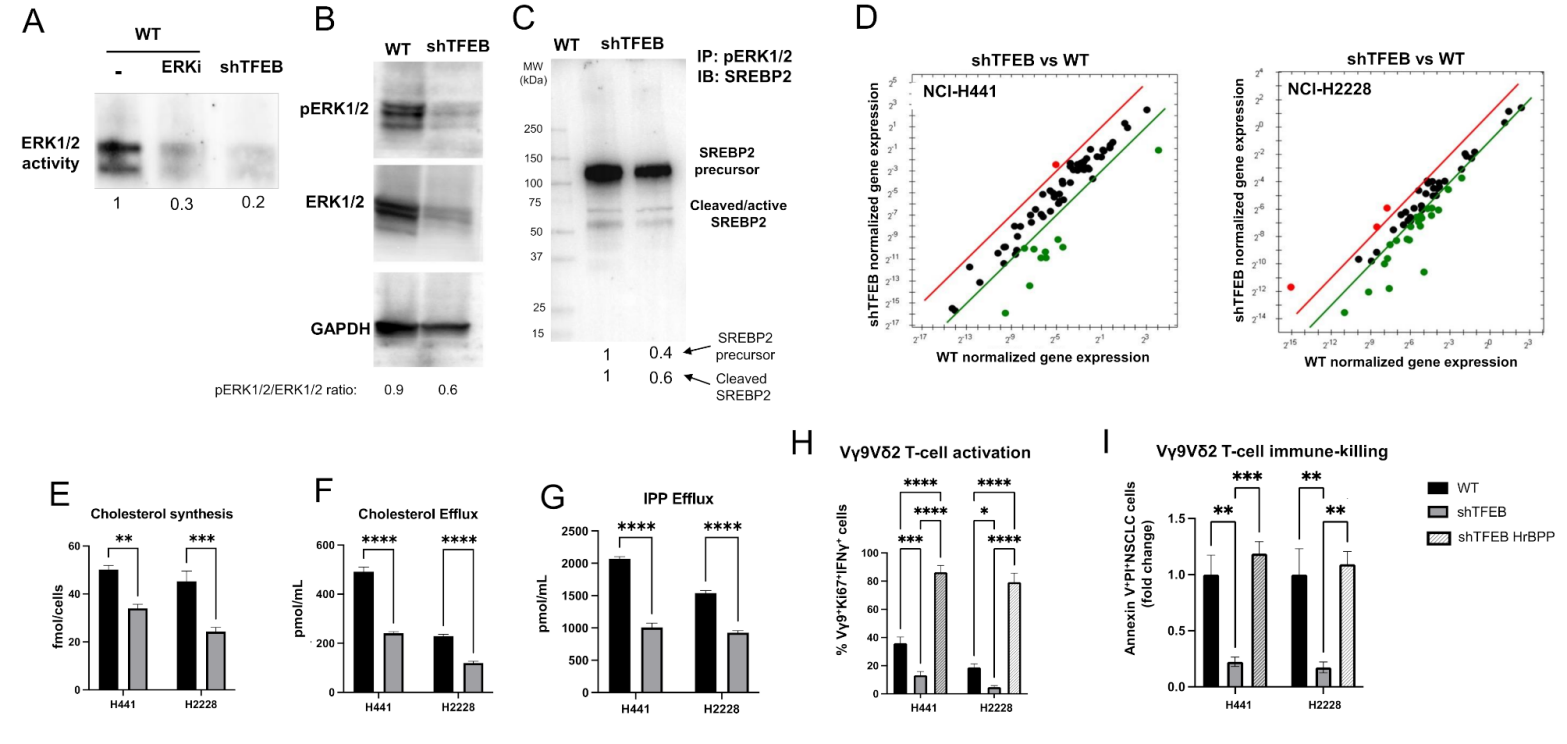
TFEB silencing modulates cholesterol homeostasis-related genes and ABCA1-mediated immuno-killing by reducing SREBP2 activation. (**A**) ERK1/2 activity, measured by a pull-down assay in wild-type (WT) and TFEB silenced (shTFEB) NCI-H2228 cells. When indicated, WT cells were pre-treated for 30 min with 1 µM of a pan-ERK inhibitor (ERKi) provided by the kit (*n* = 3 independent experiments). For the densitometric analysis, the ratio between band density in each experimental condition/band density in untreated (-) WT cells, considered 1, was calculated and reported below the figure. (**B**) NCI-H2228 cells were immunoblotted for phospho(Thr202/Tyr204)-ERK1/2, total ERK1/2 and GAPDH, used as control of equal protein loading (*n* = 3 independent experiments). The band densities of ERK1/2 and phospho(Thr202/Tyr204)-ERK1/2 are indicated below the image. The ratio pERK1/2 versus ERK1/2 was calculated as band density of pERK1/2 in each experimental condition/band density of ERK1/2 in the matched condition. The band density in untreated (-) WT cells was considered 1. (**C**) NCI-H2228 lysates were immunoprecipitated (IP) for phospho(Thr202/Tyr204)-ERK1/2 and immunoblotted (IB) for SREBP2, using an antibody recognizing both precursor and cleaved/active SREBP2 (*n* = 3 independent experiments). The density of SREBP2 precursor and cleaved SREBP2 in shTFEB cells is indicated below the image, setting the band density of SREBP2 precursor and cleaved SREBP2 in WT cells as 1. (**D**). PCR-array of cholesterol homeostasis-related genes in shTFEB-NCI-H2228 compared with WT cells (*n* = 2 independent experiments). Red dot: significantly up-regulated genes; green dot: significantly down-regulated genes. For the whole gene list: see Supplemental Table S4. (**E-G**). Cholesterol synthesis (**E**), cholesterol efflux (**F**) and IPP efflux (**G**), measured by metabolic radiolabeling in duplicates. Data are means ± SD (*n* = 3 independent experiments), in duplicates. **p < 0.01, ***p < 0.001, ****p < 0.0001: shTFEB versus WT cells. (**H**-**I**). Expansion and activation of Vγ9Vδ2 T-lymphocytes from healthy donors after co-culture with NSCLC cells, as percentage of γ9^+^Ki67^+^ IFNγ^+^ cells (**H**), and Vγ9Vδ2 T-lymphocyte-mediated NSCLC cell immuno-killing, as percentage of AnnexinV^+^PI^+^ NSCLC cells (**I**), measured by flow cytometry in duplicates. When indicated, the IPP stable analog BrHPP (IPH1101; 20 nM) was added to the culture medium. Data are means ± SD (*n* = 3 independent experiments). *p < 0.05, **p < 0.01, ***p < 0001, ****p < 0.0001: shTFEB versus WT cells

Given the pleiotropic effects of TFEB, we afterward investigated if TFEB controlled ABCA1 and ABCC1 activity by additional mechanisms, besides the transcriptional regulation. Since in melanoma TFEB silencing reduces the phosphorylation and activity of ERK1/2 [28], which in turns phosphorylates and activates the cleavage of SREBP2 [44], the main transcription factor of cholesterol homeostasis genes, we first investigated if these events occur also in NSCLC cells. ShTFEB NCI-H2228 cells had reduced ERK1/2 activity (Fig. 3A), phospho-ERK1/2 and total ERK1/2 amount (Fig. 3B). In co-immunoprecipitation experiments between ERK1/2 and SREBP2, we verified that both SREBP2 precursor and its cleaved active form interacted with phospho-ERK1/2 (Fig. 3C; Supplemental Fig. S4): in shTFEB cells, however, where the active ERK1/2 was decreased, the activation of SREBP2 was lower (Fig. 3C). Consistently, many genes involved in cholesterol uptake, synthesis and metabolism were down-regulated in shTFEB cells (Fig. 3D; Supplemental Table S4), resulting in decreased cholesterol synthesis (Fig. 3E) and efflux (Fig. 3F). Also, the efflux of IPP, transported with cholesterol by ABCA1, was lower (Fig. 3G). Since IPP is the endogenous activator of Vγ9Vδ2 T-lymphocytes, we detected a lower amount of proliferating (Ki67^+^) and activated (IFNγ^+^) Vγ9Vδ2 T-cells co-cultured with shTFEB cells (Fig. 3H). In these co-cocultures the percentage of necro-apoptotic (AnnexinV^+^PI^+^) NSCLC cells were significantly lower than in Vγ9Vδ2 T-cells/wild-type NSCLC co-cultures (Fig. 3I). The rescue of these parameters by the IPP stable analog BrHPP in silenced cells indicated that the reduced expansion/activation of Vγ9Vδ2 T-cells and immuno-killing of shTFEB cells was caused by the lower efflux of endogenous IPP via ABCA1 (Fig. 3H-I).

### TFEB silencing increases ABCC1 activity by reducing mitochondrial cholesterol and increasing ATP production via oxidative phosphorylation

**Fig. 4:**
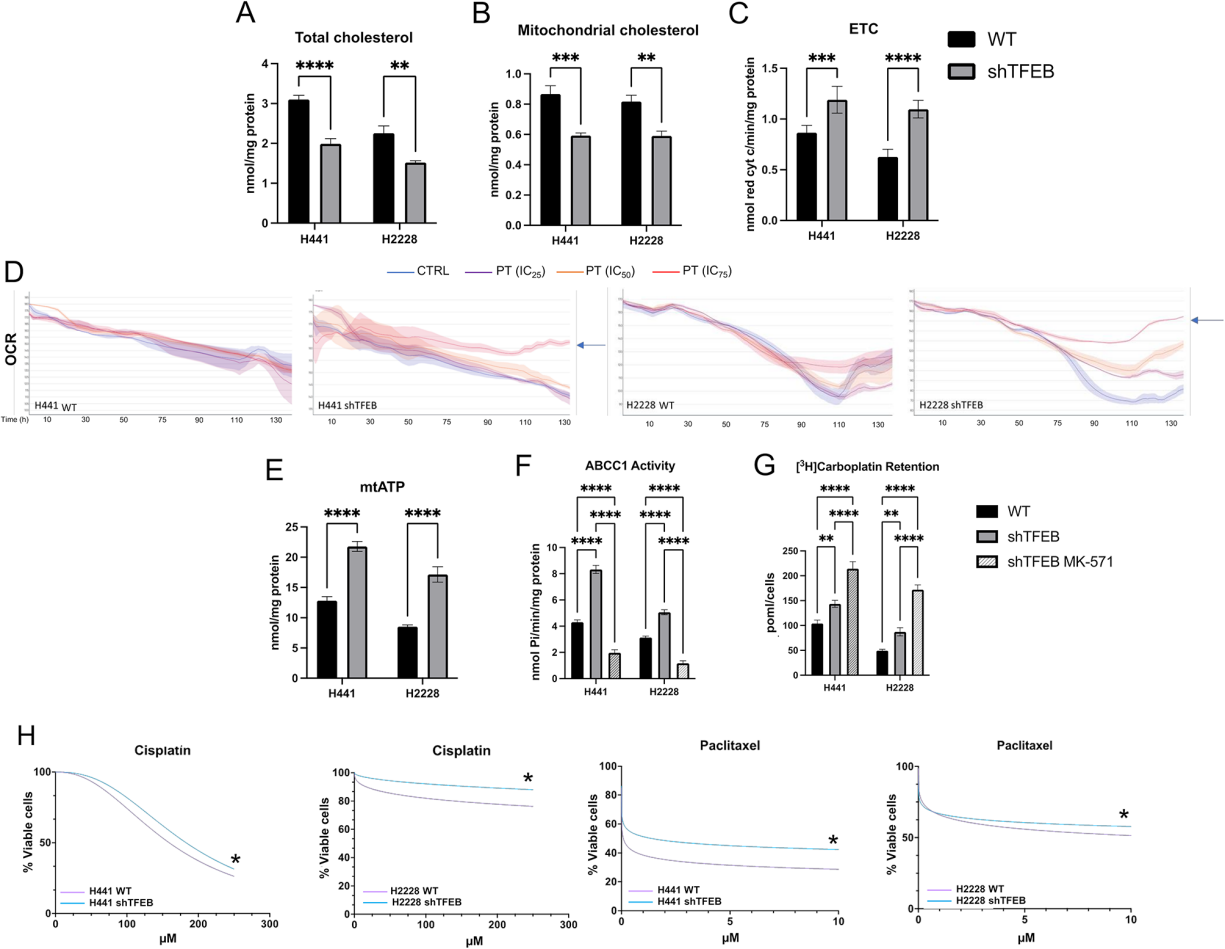
TFEB modulates ABCC1 activity by regulating mitochondrial cholesterol and metabolism. (**A-B**) Total cholesterol (**A**) and mitochondrial cholesterol (**B**) in wild-type (WT) and TFEB silenced (shTFEB) NCI-H441 and NCI-H2228, measured spectrophotometrically in duplicates. Data are means ± SD (*n* = 3 independent experiments). **p < 0.01, ***p < 0.001, ****p < 0.0001: shTFEB versus WT cells. (**C**). Electron transport chain (ETC), measured spectrophotometrically in duplicates. Data are means ± SD (*n* = 3 independent experiments). ***p < 0.001, ****p < 0.0001: shTFEB versus WT cells. (**D**). Oxygen consumption rate (OCR), monitored up to 130 h in live WT and shTFEB cells incubated without (CTRL) or with cisplatin (PT) at respective IC_25_, IC_50_ and IC_75_ for each cell lines (*n* = 3 independent experiments). (**E**) Mitochondrial (mt) ATP, measured by a chemiluminescence-based assay, in duplicates. Data are means ± SD (*n* = 3 independent experiments). ****p < 0.0001: shTFEB versus WT cells. (**F**). ABCC1 catalytic activity, measured spectrophotometrically in duplicates. When indicated, the ABCC1 inhibitor MK571 (25 µM), was added. Data are means *±* SD (*n* = 3 independent experiments). ****p < 0.0001: shTFEB versus WT cells. (**G**) Intracellular retention of [^14^C]-Carboplatin, measured after cell labelling in duplicates, in the absence or presence of MK571. Data are means ± SD (*n* = 3 independent experiments). **p < 0.01, ****p < 0.0001: shTFEB versus WT cells. (**H**) Dose-response viability in the presence of increasing concentration of cisplatin (0-250 µM) or paclitaxel (0–10 µM) for 72 h, measured in quadruplicates (*n* = 3 independent experiments). *p < 0.05: shTFEB versus WT cells

In line with the lower synthesis of cholesterol (Fig. 3E), the amount of total cholesterol in cell membranes (Fig. 4A) and mitochondrial membranes (Fig. 4B) were significantly decreased in shTFEB cells. Notably, the efficacy of ETC and the production of mitochondrial ATP via oxidative phosphorylation (OXPHOS) were impaired by a high cholesterol content in mitochondria [45]. Moreover, TFEB depletion is known to alter the assembly of complex I of ETC [46]. In keeping with these findings, the ETC flux from complex I to complex III was significantly increased in TFEB-silenced cells (Fig. 4C). The real-time OCR in parental and shTFEB cells was similar, but in the presence of a mitochondria-damaging drug as cisplatin (PT), the OCR of parental cells dropped, while the OCR of shTFEB cells remained high (Fig. 4D), indicating the preservation of OXPHOS function. Consistently with this assumption, cells silenced for TFEB had a higher production of mitochondrial ATP (Fig. 4E), which is of paramount importance in drug resistant cells because it is the main fuel of ABC transporters involved in drug efflux [47]. Indeed, shTFEB cells had strikingly increased activity of ABCC1 (Fig. 4F), coupled with decreased intracellular retention carboplatin (Fig. 4G). The platinum-retention was improved by the ABCC1 inhibitor MK571 in shTFEB cells (Fig. 4F-G), indicating that the chemotherapeutic drug was effluxed by ABCC1. Consequently, TFEB silenced cells were more resistant than parental cells to PT and paclitaxel (Fig. 4H), two drugs used in NSCLC treatment and whose poor efficacy has been related to ABCC1 expression [48, 49].

### TFEB overexpression restores sensitivity to chemotherapy and immuno-killing

**Fig. 5:**
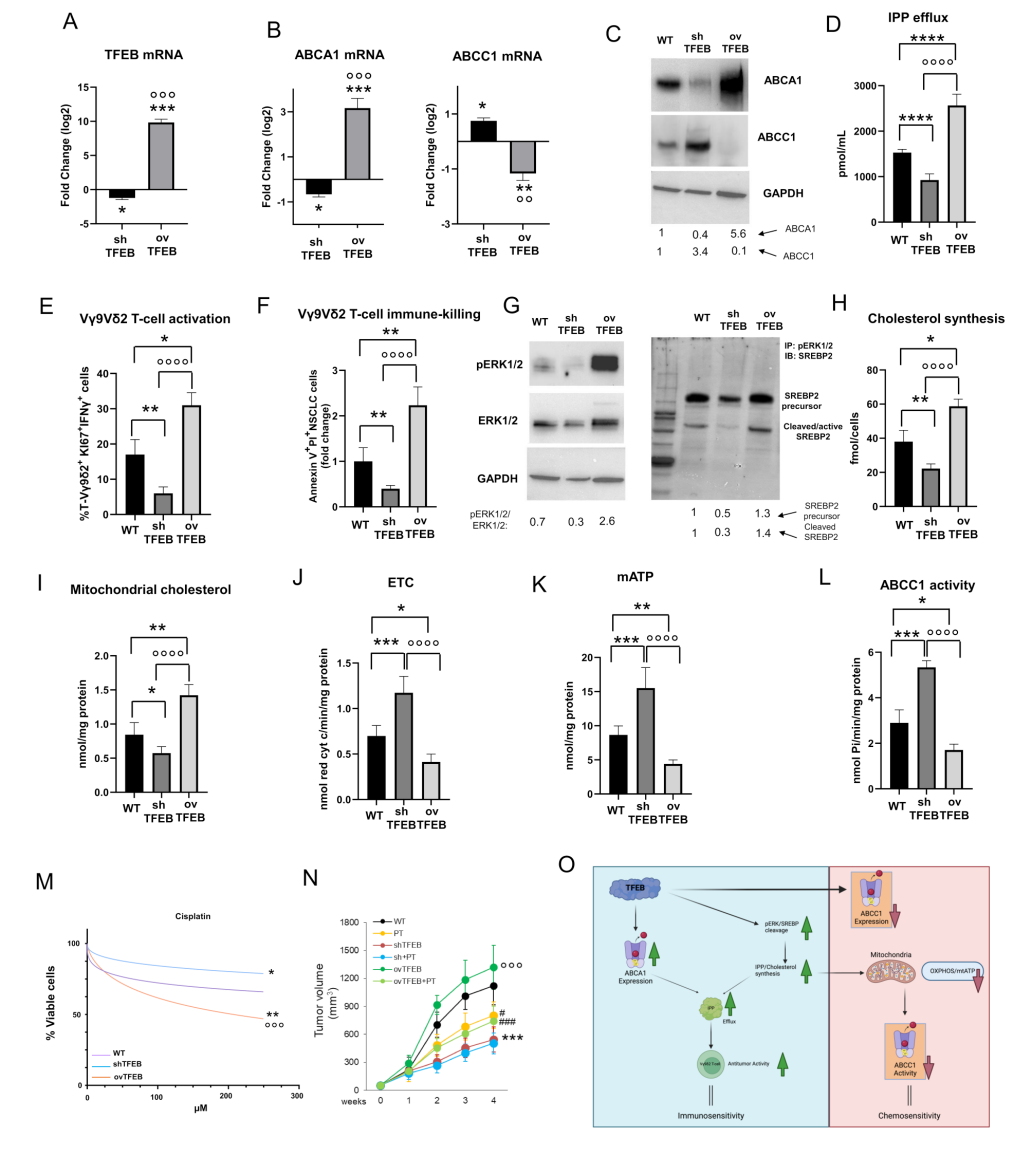
TFEB overexpression induces chemo- and immuno-sensitization of non-small cell lung cancer cells. Wild-type NCI-H2228 cells were transduced with the pLKO empty vector (WT), silenced for TFEB (shTFEB), silenced for TFEB and then transduced with the vector overexpressing the constitutively active TFEBS142A (ovTFEB). (**A-B**) TFEB, ABCA1 and ABCC1 mRNAs measured in triplicates. Data are means ± SD of the mRNA fold change (*n* = 3 independent experiments). *p < 0.05, **p < 0.01, ***p < 0.001: shTFEB/ovTFEB versus WT cells; °°*p* < 0.01, °°°*p* < 0.001: ovTFEB vs. shTFEB cells. (**C**) Immunoblot of ABCA1 and ABCC1. GAPDH was used as control of equal protein loading (*n* = 3 independent experiments). The band density of ABCA1 and ABCC1 is indicated below the image. (**D-F**) IPP efflux, expansion/activation of Vγ9Vδ2 T-lymphocytes, Vγ9Vδ2 T-lymphocyte-mediated NSCLC cell immuno-killing, measured in duplicates. Data are means ± SD (*n* = 3 independent experiments). *p < 0.05, **p < 0.01, ****p < 0.0001: shTFEB/ovTFEB versus WT cells; °°°°*p* < 0.0001: ovTFEB vs. shTFEB cells. (**G**) Immunoblot of pERK1/2, total ERK1/2, immunoprecipitation (IP) of phospho(Thr202/Tyr204) followed by immunoblotting (IB) for SREBP2, using an antibody recognizing both precursor and cleaved/active SREBP2. GAPDH was used as control of equal protein loading (*n* = 3 independent experiments). The density ratio between phospho(Thr202/Tyr204) ERK1/2 versus ERK1/2 (setting the band density of ERK1/2 in WT cells as 1), the density of SREBP2 precursor and cleaved SREBP2 (setting the band density of SREBP2 precursor and cleaved SREBP2 in WT cells as 1) are indicated below the image. (**H-I**) Cholesterol synthesis and mitochondrial cholesterol measured in duplicates. Data are means ± SD (*n* = 3 independent experiments). *p < 0.05, **p < 0.01: shTFEB/ovTFEB versus WT cells; °°°°*p* < 0.0001: ovTFEB vs. shTFEB cells. (**J-K**) Mitochondrial ETC and ATP, measured in duplicates. Data are means *±* SD (*n* = 3 independent experiments). *p < 0.05, **p < 0.01, ***p < 0.001: shTFEB/ovTFEB versus WT cells; °°°°*p* < 0.0001: ovTFEB vs. shTFEB cells. (**L**) ABCC1 catalytic activity, measured in duplicates. Data are means ± SD (*n* = 3 independent experiments). *p < 0.05, ***p < 0.001: shTFEB/ovTFEB versus WT cells; °°°°: ovTFEB vs. shTFEB cells. (**M**) Dose-response viability in the presence of increasing concentration of cisplatin (0-250 µM) for 72 h, measured in quadruplicates. Data are means ± SD (*n* = 3 independent experiments). *p < 0.05,**p < 0.01: shTFEB/ovTFEB versus WT cells; °°°*p* < 0.05: ovTFEB vs. shTFEB cells. (**N**) Tumor growth, measured by a caliper, of 1 × 10^6^ NCI-H2228 wild-type (WT), TFEB-silenced (shTFEB) and shTFEB cells overexpressing TFEBS142A (ovTFEB), implanted subcutaneously in female NOD SCID-γ (NSG) mice engrafted with human hematopoietic CD34^+^ cells (Hu-CD34^+^). When tumors reached the volume of 50 mm^3^, animals (*n* = 5/group) were randomized and treated for 3 weeks as it follows: (1) control (CTRL) group, treated with 0.1 mL saline solution intravenously (i.v.), once a week; (2) cisplatin (PT) group, treated with 2 mg/kg cisplatin i.v., once a week. Data are means *±* SD. ***p < 0.001: CTRL shTFEB versus WT; °°°*p* < 0.001: CTRL ovTFEB versus shTFEB; ^#^*p* < 0.05, ^###^*p* < 0.001: PT-treated tumor vs. corresponding CTRL. (**O**) Scheme of the proposed working model. TFEB induces ABCA1 and decreases ABCC1, modulating both the transcription and the activity of these transporters, controlling chemo- and immuno-sensitivity in NSCLC cells (created with BioRender.com)

As proof of concept that TFEB is a controller of chemo- and immuno-resistance in NSCLC cells, we verified if the overexpression of the constitutively active TFEBS142A form in cells previously silenced for endogenous TFEB (ovTFEB; Fig. 5A) may rescue the phenotype produced by TFEB silencing. The overexpression of TFEB increased ABCA1 and decreased ABCC1 at mRNA (Fig. 5B) and protein (Fig. 5C) level. According to the increased amount of ABCA1, the efflux of IPP was also increased (Fig. 5D), producing a higher expansion and activation of Vγ9Vδ2 T-lymphocytes (Fig. 5E), coupled with higher Vγ9Vδ2 T-lymphocyte immuno-killing of overTFEB-cells (Fig. 5F). Furthermore, the overexpression of TFEB increased pERK1/2 and SREBP2 activation (Fig. 5G), cholesterol synthesis (Fig. 5H) and cholesterol incorporation in mitochondria (Fig. 5I), determining a decrease in ETC (Fig. 5J) and mitochondrial ATP production (Fig. 5K). The lower availability of ATP decreased the catalytic activity of ABCC1 (Fig. 5L) and increased the sensitivity of ovTFEB cells to PT (Fig. 5M).

To validate the data obtained in vitro, we implanted WT, shTFEB and ovTFEB NCI-H2228 cells in immunocompetent Hu-CD34^+^ NSG mice. The levels of TFEB were verified in the explanted tumors by RT-PCR (Supplemental Fig. S5). In line with previous findings obtained in melanoma [28], shTFEB tumors grew less than WT tumors. By contrast, ovTFEB tumors had a higher rate of growth. When treated with PT, shTFEB tumors were more resistant and ovTFEB tumors were more sensitive than WT tumors (Fig. 5N).

Overall, our data suggest that TFEB has a multifaceted role on chemo- and immuno-sensitivity in NSCLC. TFEB silencing decreased IPP efflux, ABCA1 expression and activity, preventing the immuno-killing mediated by Vγ9Vδ2 T-lymphocytes. At the same time, it transcriptionally down-regulated ABCC1 and decreased its catalytic activity fueled by the OXPHOS-derived mitochondrial ATP, two events that can be consequent to the increased amount of mitochondrial cholesterol (Fig. 5O). Hence, TFEB can be considered a chemo-and immuno-sensitizer factor.

### Tuning cholesterol homeostasis by zoledronic acid chemo-immuno-sensitizes non-small cell lung cancers with low TFEB

Since TFEB silencing induced chemo-immuno-resistance in NSCLC cells, a potential strategy to re-sensitize cells with low TFEB to PT and Vγ9Vδ2 T-lymphocytes killing, could be increasing the amount of IPP without reducing the amount of cholesterol. To this aim, we used zoledronic acid, an aminobisphosphonate that inhibits farnesyl pyrophosphate synthase (FPPS), the enzyme downstream IPP production in the cholesterol synthesis [50]. Zoledronic acid increases the IPP accumulation and its efflux through ABCA1, promoting the expansion of Vγ9Vδ2 T-lymphocytes [9]. At the same time, since it acts several steps downstream the pacemaker enzyme of cholesterol synthesis, 3-β-hydroxy-3β-methyl-glutaryl-coenzyme A reductase, at low concentration it may increase IPP without decreasing significantly cholesterol. Moreover, to maximize the tumor targeting, limiting the uptake of the aminobisphosphonate by the bone, we used a self-assembled lipid nanoformulation of zoledronic acid (NZ), previously reported to have a better tumor-to-bone ratio than the free drug [51]. In preliminary experiments on NCI-H2228 cells, we verified that 100 nM NZ did not reduce the cholesterol synthesis in shTFEB cells (Supplemental Fig. S6A), but it increased the IPP synthesis (Supplemental Fig. 6B) and efflux (Supplemental Fig. 6C), the percentage of activated Vγ9Vδ2 T-lymphocytes (Supplemental Fig. 6D) and the tumor cell immuno-killing by Vγ9Vδ2 T-lymphocytes, both alone and in combination with PT (Supplemental Fig. 6E). Moreover, NZ did not decrease mitochondrial cholesterol in TFEB-silenced cells compared to untreated cells (Supplemental Fig. 6F), nor increased ETC (Supplemental Fig. 6G), mitochondrial ATP (Supplemental Fig. 6H) and ABCC1 activity (Supplemental Fig. 6I). Carboplatin retention was also unchanged compared to shTFEB cells (Supplemental Fig. 6J).

**Fig. 6:**
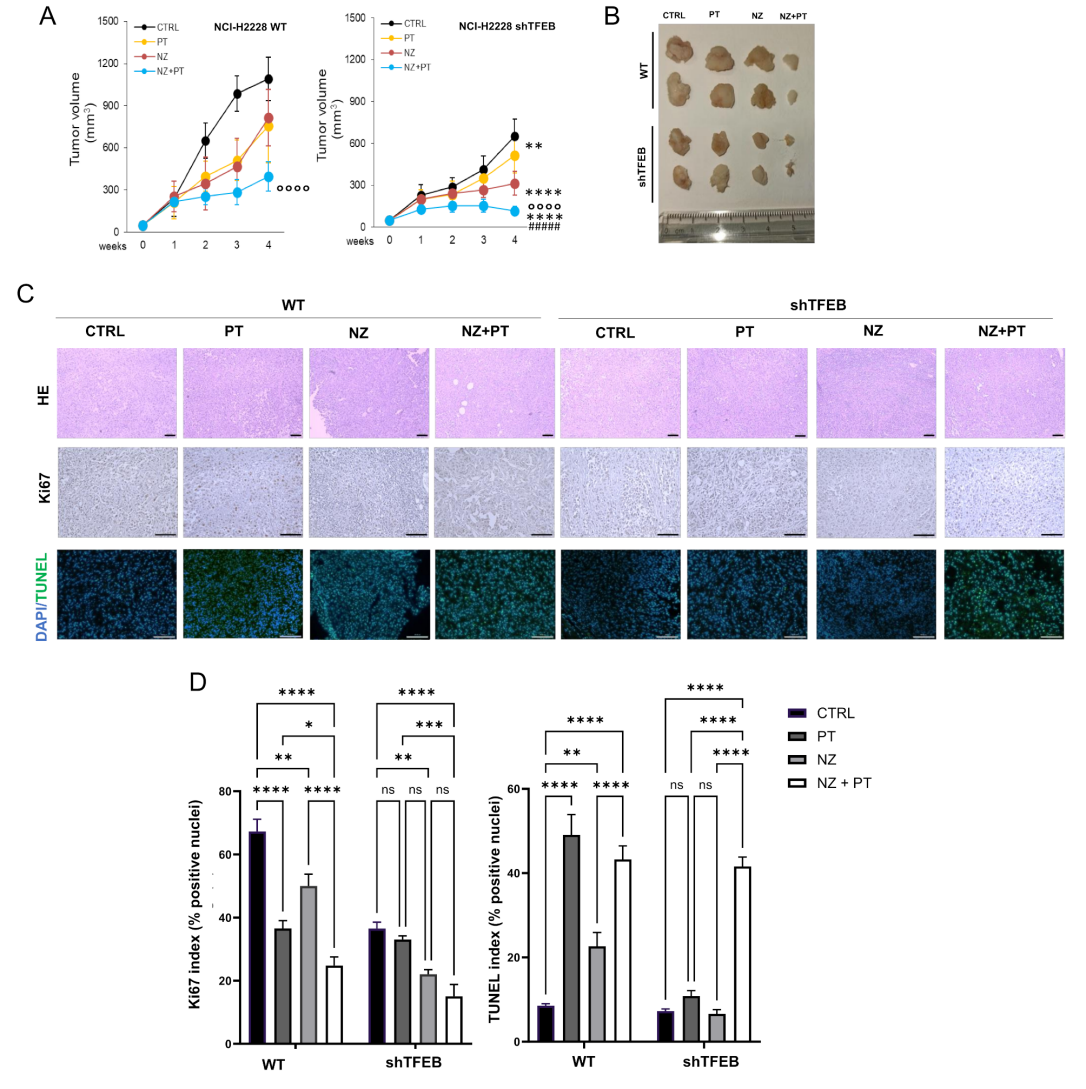
Self-assembled zoledronic acid nanoparticles restore chemosensitization in TFEB-silenced non-small cell lung cancers. (**A-B**). 1 × 10^6^ NCI-H2228 wild-type (WT) and TFEB-silenced (shTFEB) cells were injected subcutaneously in female Hu-CD34^+^ NSG mice. When tumors reached the volume of 50 mm^3^, animals (*n* = 5/group) were randomized and treated for 3 weeks as it follows: (1) control (CTRL) group, treated with 0.1 mL saline solution intravenously (i.v.), once a week; (2) cisplatin (PT) group, treated with 2 mg/kg cisplatin i.v., once a week; (3) NZ, treated with 1 mg/kg NZ i.v., once a week; (4) NZ + cisplatin group, receiving both drugs i.v., once a week simultaneously. Animals were euthanized on day 28 after randomization with zolazepam (0.2 mLl/kg) and xylazine (16 mg/kg). (**A**) Tumors growth was monitored by a caliper. Data are means ± SD. **p < 0.01, ****p < 0.0001: shTFEB versus WT tumors; ^°°°^*p* < 0.0001: NZ + PT versus CTRL (WT or shTFEB); ^####^*p* < 0.0001: NZ + PT versus PT alone (WT or shTFEB). (**B**) Representative photos of excised tumors. (**C**) Hematoxylin-eosin (HE), Ki67 and TUNEL staining of tumor sections from each group (5 field/each tumor). Objective: 10× (HE) or 20× (Ki67, TUNEL); ocular: 10×. Bar = 100 μm. (**D**) Ki67 and TUNEL quantification (% positive nuclei over 100 nuclei analyzed), in 5 fields/sample. Data are means ± SD. *p < 0.05, **p < 0.01, ***p < 0.001, ****p < 0.0001

To demonstrate that NZ could be a good adjunctive agent against TFEB lowly expressing chemo-immuno-resistant tumors, we implanted wild-type and shTFEB NCI-H2228 cells in Hu-CD34^+^ NSG mice, bearing different human T-lymphocyte lineages including Vγ9Vδ2 T-lymphocytes [52]. The analysis of TFEB RNA from tumors extracts indicated the persistence of TFEB silencing in all treatment groups (Supplemental Fig. S7). As previously anticipated, shTFEB tumors were less sensitive to PT than wild-type ones (Fig. 6A-B). NZ alone delayed tumor growth but it did not reduce the tumor volume at our end point in wild-type xenografts. Conversely, it markedly decreased tumor volume in shTFEB xenografts. Notably, in both wild-type and shTFEB tumors the combination of NZ + PT strongly decreased tumor growth: while in wild-type xenografts we observed a lower tumor growth, in shTFEB xenografts we even achieved a tumor regression (Fig. 6A-B).

PT reduced intratumor proliferation and increased apoptosis in wild-type but not in shTFEB tumors. Notably, the combination NZ + PT rescued the pro-apoptotic effect of PT in shTFEB tumors (Fig. 6C-D). The combination treatments did not show relevant signs of systemic toxicity: the *post-mortem* pathological analysis did not reveal appreciable histological alterations in heart, liver, lung, kidney and spleen in each experimental group (Supplemental Fig. S8). Similarly, the hematological and chemical parameters measured immediately after euthanasia indicated no signs of toxicity for bone marrow (RBC, Hb, WBC, PLT), liver (LDH, AST, ALT, AP), kidney (creatinine), muscles and heart (CPK) in each group of treatment (Supplemental Table S5).

### Zoledronic acid rescues cisplatin-induced intratumor apoptosis and reshapes the tumor immune-environment of shTFEB tumors increasing anti-tumor populations

**Fig. 7:**
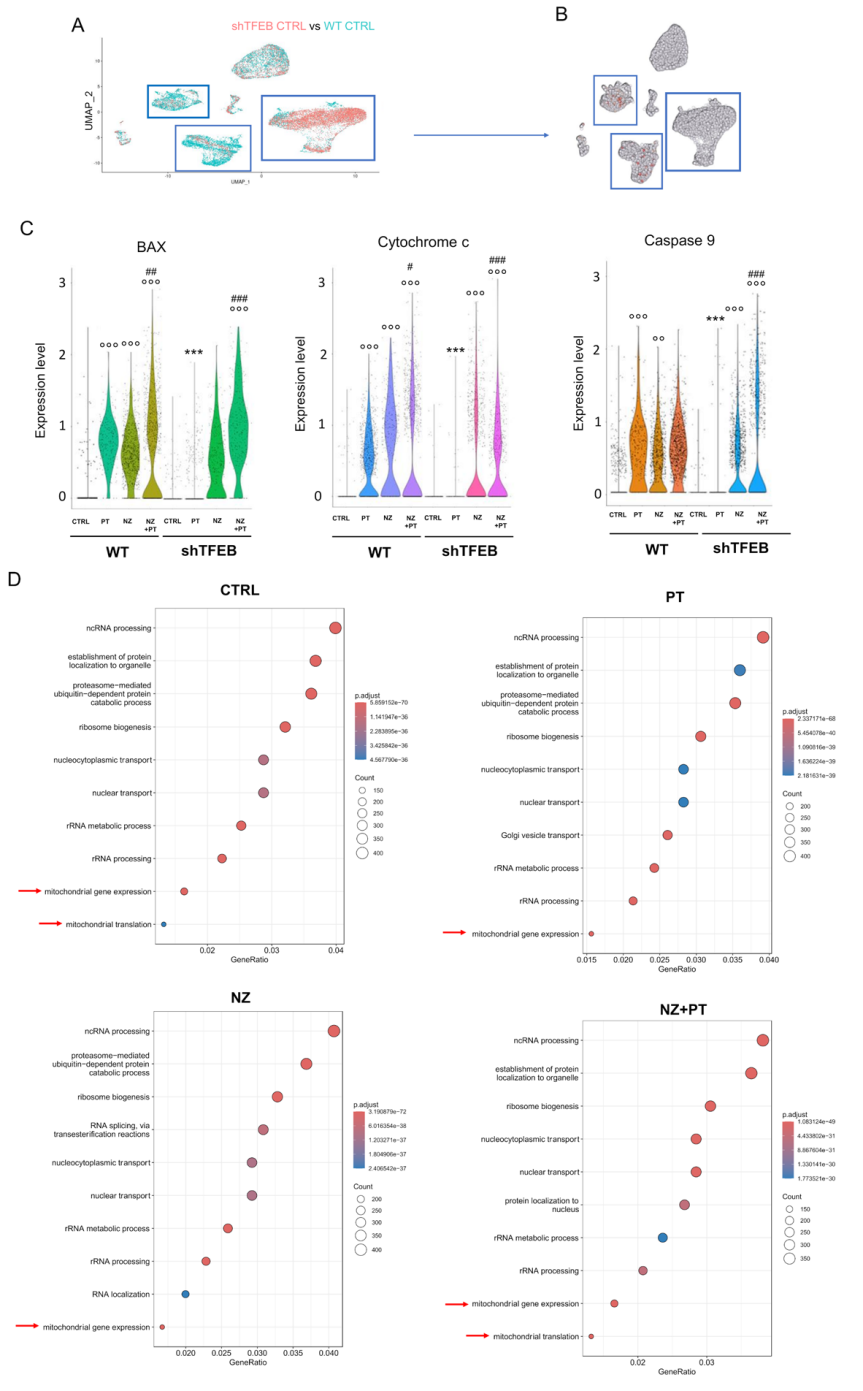
Self-assembled zoledronic acid nanoparticles induce a pro-apoptotic signature in TFEB-silenced tumors. (**A**) UMAP visualization of cell populations differentially present in WT CTRL versus shTFEB CTRL tumors (see Methods section for the experimental prototol). Cancer cell clusters are indicated by the blue squares. (**B**) Comparison of the TFEB-hot spots in WT CTRL tumors versus shTFEB CTRL tumors, in the 3 tumor clusters identified in panel A (blue squares). (**C**) Violin plots of differentially expressed pro-apoptotic genes BAX, cytochrome c and caspase 9, emerged by the scRNA-Seq analysis. ***p < 0.0001: shTFEB versus corresponding condition in WT tumors; ^°°^*p* < 0.01, ^°°°^*p* < 0.001: PT, NZ or NZ + PT versus CTRL (WT or shTFEB); ^#^*p* < 0.05, ^##^*p* < 0.01, ^###^*p* < 0.001: NZ + PT versus PT alone (WT or shTFEB). **D.** GSEA results: top-10 gene categories in clustered shTFEB cancer cells vs. WT cancer cells. Red arrows: mitochondria-related categories

The silencing of TFEB also changed the susceptibility to apoptosis of cancer cells and the tumor immune micro-environment (TIME) qualitative and quantitative composition, as indicated by the scRNA-Seq analysis of the immuno-xenografts. Three clusters were identified as cancer cells (Fig. 7A). In two of them, TFEB was higher in WT tumors than in shTFEB tumors (Fig. 7B), confirming the persistence of the TFEB^low^ phenotype. By comparing WT and shTFEB tumors clusters (vehicle group) for the differentially expressed pro-apoptotic genes, we found a mini-signature (*BAX*, *cytochrome c*, *caspase 9*) more expressed in WT than in silenced tumors upon treatment with PT (Fig. 7C). The combination of NZ + PT induced a marked increase in the expression of the three apoptotic genes in wild-type as well as shTFEB tumors (Fig. 7C), supporting the previous observations that the antitumor effect of the NZ + PT combination was caused by increased intratumor apoptosis (Fig. 6C-D).

Among the top-10 gene categories enriched in shTFEB clustered cancer cells, we found the “mitochondrial gene expression” (in all the treatment groups) and “mitochondrial translation” (in vehicle-treated and NZ + PT-treated group; Fig. 7C) categories, that may explain the higher mitochondrial metabolism observed in shTFEB cells.

**Fig. 8:**
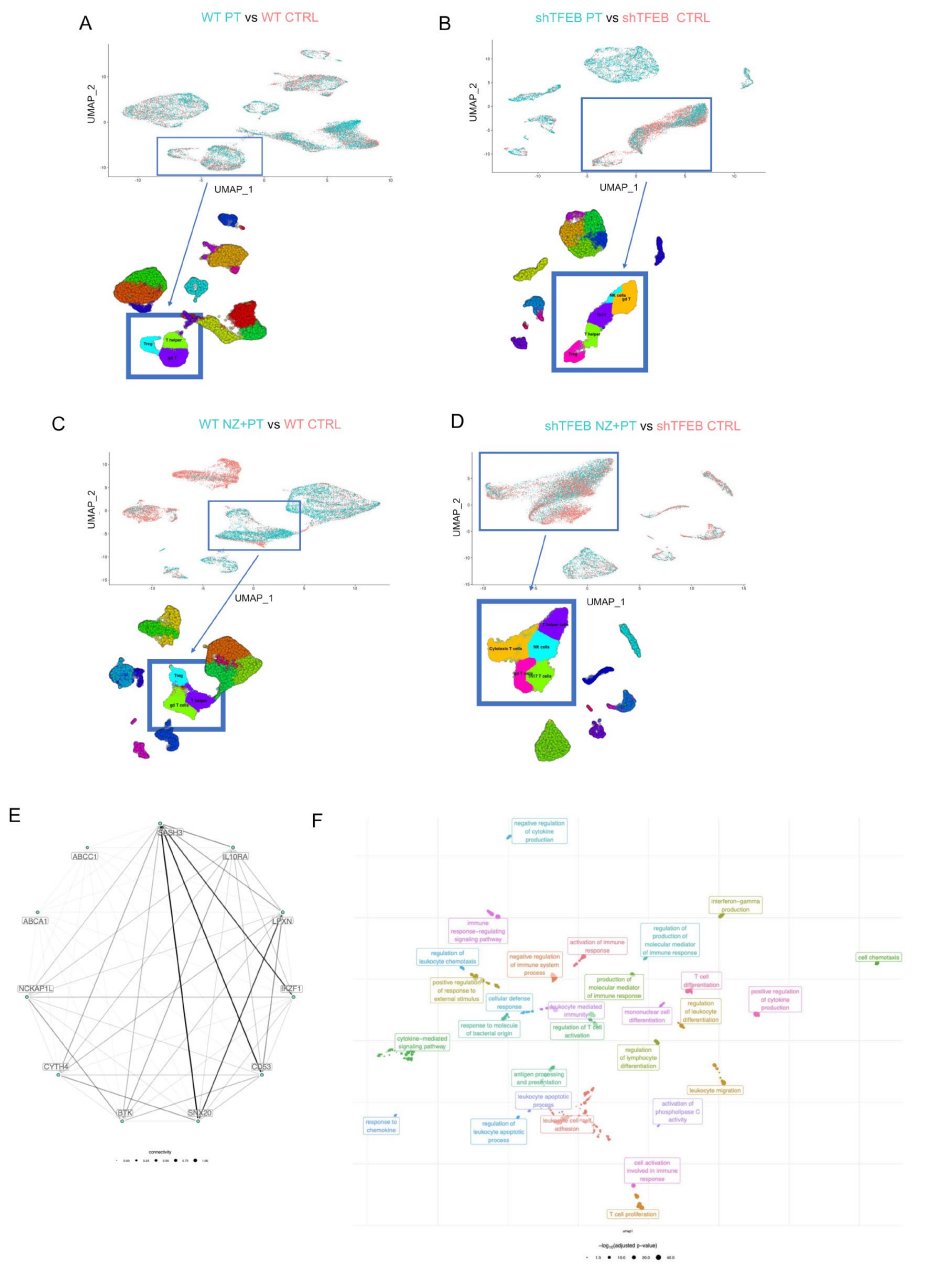
TFEB-silenced non-small cell lung cancers have an immune-evasive environment, rewired by self-assembled zoledronic acid nanoparticles. UMAP of the differentially present immune-infiltrating populations, identified by scRNA-Seq analysis, in the following comparisons: WT PT versus WT CTRL (**A**), shTFEB PT versus shTFEB CTRL (**B**), WT NZ + PT versus WT CTRL (**C**), shTFEB NZ + PT versus shTFEB CTRL (**D**; *n* = 2 tumors/group). (**E**). Top down-regulated gene network in TFEB^low^ABCA1^low^ABCC1^high^ patients of the TGCA-LUAD cohort (*n* = 531 patients). (**F**) Biological processes associated to the gene network of panel E

As far as the TIME is concerned, wild-type tumors had significantly up-regulated categories related positive regulation of immune system response, leukocyte and T-cell activation, immune response-activating signal transduction (Supplemental Figure S9), indicating an anti-tumor immune-environment stronger in wild-type than in shTFEB tumor. Upon PT treatment, the processes related to the immune activation were higher in wild-type than in shTFEB tumors (Supplemental Fig. S10A-B). The scRNA-Seq analysis of the immune-populations indicated that in wild-type tumors treated with PT CD4^+^T-helper lymphocytes and Vγ9Vδ2 T-lymphocytes (identified as cells expressing T cell receptor delta constant *TRDC* gene [53]), and pro-tumoral T-regulatory (Treg) cells were differentially detected (*p* < 0.05) compared with vehicle-treated group (Fig. 8A). While CD4^+^T-helper lymphocytes were downregulated and Vγ9Vδ2 T-lymphocytes were minimally increased, Treg cells were hugely augmented (Supplemental Fig. S10C). The TIME of shTFEB tumors was more heterogeneous in composition upon PT treatment, including – among the differentially detected populations - anti-tumor (CD4^+^T-helper lymphocytes, Vγ9Vδ2 T-lymphocytes and natural killer - NK) cells, as well as pro-tumor (Treg and Th17 T-lymphocytes) cells (Fig. 8B). All these populations were down-regulated in PT-treated shTFEB tumors (S10), making controversial the anti-tumor or pro-tumor effect of PT on TIME of silenced tumors.

The treatment with NZ + PT increased the number of the up-regulated immune-activating processes, in wild-type tumors and particularly in shTFEB tumors (Supplemental Fig. S11A-B). Consistently, according to the scRNA-Seq analysis, this combination increased the amount of Vγ9Vδ2 T-cells, but also of CD4^+^T-helper lymphocyte and Treg cells in wild-type tumors (Fig. 8C; Supplemental Fig. S11C). Intriguingly, in shTFEB tumors, the combination of NZ + PT significantly expanded the Vγ9Vδ2 T-lymphocytes and other anti-tumor populations as CD4^+^T-helper lymphocytes, NK cells and CD8^+^T-cytotoxic lymphocytes that were not detected in the TME of vehicle-treated shTFEB tumors, while it decreased Treg cells (Fig. 8D; Supplemental Fig. S11D), suggesting a reshaping toward a less immuno-suppressive TIME.

To clarify how much TIME composition impacts on PT-resistance in silenced tumors, we implanted wild-type and shTFEB tumors in immuno-deficient NSG mice: also, in these mice shTFEB tumors grew less and were less sensitive to PT than wild-type tumors (Supplemental Fig. S12A). The combination of NZ + PT decreased tumor growth in wild-tipe tumor, with a very limited effects in NSG mice (Supplemental Fig. S12A) compared to the effects achieved in Hu-CD34^+^ mice (Fig. 6A). In terms of tumor volume reduction, the effect of NZ + PT was greater in shTFEB tumors than WT tumors implanted Hu-CD34^+^ mice (final tumor volume: 24.15 ± 3.97 in shTFEB tumors versus 39.09 ± 4.57 in WT tumors), while it did not differ for tumors implanted in NSG mice (Supplemental Fig. S12B). These data suggested that TIME composition is critical to determine the response to PT in shTFEB tumors: the rewiring of the immunosuppressive TIME typically associated with these tumors toward an anti-tumor TIME was also paralleled by the rescue of PT efficacy.

The more immuno-evasive profile of shTFEB tumors was confirmed by the WGCNA performed on TFEB^high^ and TFEB^low^ tumors of the TCGA-LUAD dataset. Indeed, among the modules significantly down-regulated in TFEB^low^ABCA1^low^ABCC1^high^ phenotype there was the gene network shown in Fig. 8E, including genes associated to biological process as chemotaxis, regulation of immune response, antigen processing and presentation, activation and differentiation of T-cells and cytokine production (Fig. 8F).

## Discussion

Drug resistance, together with low immuno-killing by the host immune system, are the main causes of the limited success of systemic treatment in NSCLC patients. More than 30 years passed from the development of the first inhibitors of ABC transporters, key players in drug resistance, but many attempts failed in the context of clinical trials, because of poor specificity and high toxicity of the inhibitors. These serial failures suggested the need for new approaches, e.g. targeting the metabolic and molecular circuitries controlling ABC transporters [54, 55]. In this work we dissected the role of TFEB on the regulation of ABC transporters involved in the response to chemotherapy and to Vγ9Vδ2 T-lymphocyte killing, with the aim to find new chemo-immuno-sensitizing approaches for NSCLC.

First, we evaluated if TFEB and the ABC transporters of interest (the drug resistance inducer ABCC1 and the immuno-sensitizer protein ABCA1) were correlated and had a clinical relevance in NSCLC patients. An increased level of TFEB, along with LAMP2a and Cathepsin D, has been correlated with poor prognosis in NSCLC patients [56], in contrast with our results. However, the findings depicting TFEB as a negative prognostic factor were obtained on squamous cell lung carcinoma, while in our analysis of TCGA and retrospective cohort, only adenocarcinomas were considered. We cannot exclude that TFEB had different prognostic meaning in different histological NSCLC.

ABCC1 was found higher in chemo-naïve NSCLC tumors compared to normal tissue [57], and was correlated with poor prognosis [58]. ABCA1 was down-regulated by miRNA-200b-3, an inducer of proliferation and metastasis in NSCLC [59], indirectly suggesting that high levels ABCA1 might have a positive biological implication in NSCLC. Our analysis of the TCGA-LUAD cohort indicated that patients with low TFEB, low ABCA1 and high ABCC1 had worse OS. Since TFEB and ABC transporters co-exist in patients, we examined the impact of their associations. We found that the TFEB^low^ABCA1^low^ABCC1^high^ phenotype is associated with the poorest OS, while the TFEB^high^ABCA1^high^ABCC1^low^ phenotype had the best OS. These data were validated in two cohorts of patients receiving chemotherapy and immune checkpoint inhibitors as first-line treatment, at our institution: in both groups high TFEB, high ABCA1 and low ABCC1 were independent positive predictive and prognostic factors. The TFEB^high^ABCA1^high^ABCC1^low^ phenotype was associated with significantly better PFS and OS, suggesting that this phenotype implies a better response to chemo- and immunotherapy in NSCLC patients.

The involvement of TFEB in PT resistance has been already reported. Indeed, it is well known that TFEB promotes lysosomal biogenesis and a consequent sequestration of chemotherapeutic drugs including PT within lysosomes [60]. This mechanism is documented in tongue squamous cell carcinoma [61]. The lack of colocalization of ABCC1 with lysosomes in our experimental models and the downregulation of ATP7B, a TFEB-target gene that sequesters PT within lysosomes, however, exclude that the main mechanism of PT resistance induced by TFEB is the drug sequestration within lysosome. Indeed, silenced cells were more resistant to PT despite the localization of ABCC1 outside the lysosomal compartment and the lower levels of ATP7B.

To investigate the mechanisms underlying the change in chemosensitivity, we analyzed a set of 6 NSCLC lines, expressing different levels of ABCA1 and ABCC1 on cell surface, and we silenced TFEB in the two cell lines with the highest endogenous levels. Interestingly, both silenced cell lines had a down-regulation of ABCA1 and an upregulation of ABCC1. To the best of our knowledge, this is the first time that TFEB was correlated with the differential expression of ABC transporters.

TFEB acted at transcriptional level, without changing post-translational modifications as ubiquitination. The overexpression of TFEB in shTFEB cells produced the opposite pattern in terms of ABC transporters (ABCA1 upregulation and ABCC1 downregulation), proving that TFEB is a direct controller of the transcription of these two genes. We are aware that TFEB overexpression may also impact on endocytosis, recycling and autophagy of several membrane proteins, in an unspecific way [62]. However, the effects of TFEB seem independent from these processes, because ABC transporters were not localized in the endosomal/lysosomal compartment in our cell models.

Subsequently, we investigated if TFEB also regulated the activity of these transporters, beyond their expression. TFEB controls the lysosomal degradation of neutral lipids, including cholesterol [18, 63]. Furthermore, previous microarrays showed that TFEB modulates the expression of genes involved in sterols and isoprenoids synthesis [63]. Cholesterol and its isoprenoid precursor IPP are endogenous substrates of ABCA1, and IPP efflux via ABCA1 is the driver of Vγ9Vδ2 T-lymphocyte-mediated immuno-killing of cancer cells [9–11]. In NSCLC silenced for TFEB, several genes involved in cholesterol synthesis, under the transcriptional control of SREBP2, were downregulated. Notably, the cleavage, nuclear translocation and activation of SREBP2 is induced by ERK1/2 that directly phosphorylates the protein [44]. In line with data already reported in melanoma cells [28], we found that shTFEB NSCLC cells had decreased activity and expression of ERK1/2. Co-immunoprecipitation assays demonstrated that pERK1/2 and SREBP2 physically interact. This interaction was lower in shTFEB cells that had decreased cleavage of the transcriptionally active fragment of SREBP2, justifying the global down-regulation of genes involved in cholesterol homeostasis. The molecular circuitry TFEB/SREPB2/cholesterol homeostasis genes is not cell type or cancer type-specific: indeed, THP-1 macrophages exposed to reactive oxygen species had increased nuclear translocation of TFEB, increased endogenous synthesis of cholesterol and efflux via ABCA1 [64]. Beside lower synthesis, shTFEB silenced NSCLC cells had lower effluxes of cholesterol and IPP. This event, together with the lower transcription of ABCA1, explained the reduced activation and immuno-killing of Vγ9Vδ2 T-cells co-cultured with NSCLC cells.

Recently it has been shown that cholesterol is a constituent of mitochondrial membranes, where it impairs the efficiency of OXPHOS if present at high levels [45, 65]. In addition, it has been reported that TFEB depletion impairs ETC flux [46]. Hence, high cholesterol content in mitochondria and TFEB depletion can determine a mitochondrial ATP crash. Besides representing a key source of energy, mitochondrial ATP generated by OXPHOS is the main fuel of ABC transporters involved in drug efflux [47]. Interestingly, TFEB-silenced cells had lower levels of mitochondrial cholesterol, coupled with higher ETC flux, mitochondrial ATP production and ABCC1 activity, yielding a decreased retention of carboplatin. These events, together with the transcriptional upregulation of ABCC1, explain the chemoresistance induced by TFEB silencing. In addition, shTFEB cells treated with PT did not suffer a drop in OCR, differently from wild-type cells: this means a preserved OXPHOS and ATP production, even in the presence of chemotherapeutic drugs. Since part of the toxic effect of PT is caused by mitochondrial damage [66], the preserved efficiency of OXPHOS in TFEB-silenced cells represents an additional factor determining chemoresistance. Notably, the overexpression of TFEB increased the synthesis of IPP and cholesterol, restoring the IPP/Vγ9Vδ2 T-cell-dependent immuno-killing, and the mitochondrial cholesterol/OXPHOS/ATP-dependent chemoresistance, acting instead as a chemo- and immuno-sensitizing factor in vitro and in vivo.

Since the synthesis of the cholesterol upstream metabolite IPP promotes immuno-killing, but low levels of cholesterol trigger chemoresistance, we tried to disrupt this metabolic balance by setting up a pharmacological strategy that increases IPP without varying cholesterol levels, with the goal of achieving a chemo-immuno-sensitization of NSCLC tumors with low levels of TFEB. To this aim, we used nanoparticles encapsulating zoledronic acid (NZ), an inhibitor of FPPS, i.e. the enzyme that catabolizes IPP: at the low concentration selected in our experimental model, NZ did not affect cholesterol synthesis, but it was sufficient to induce an accumulation of intracellular IPP coupled with its increased efflux. After verifying in vitro that NZ increased Vγ9Vδ2 T-cell-dependent immuno-killing, without increasing OXPHOS, mitochondrial ATP and ABCC1 activity, we validated our strategy in Hu-CD34^+^ NSG mice bearing wild-type or shTFEB-NCI-H2228 xenografts. shTFEB NSCLC tumors were less sensitive to PT than wild-type tumors and the chemosensitivity was rescued only when the chemotherapeutic drug was administered with NZ. In melanoma, TFEB silencing markedly impaired cell cycle [28]: this phenotype was mirrored by shTFEB NSCLC tumors that had also a decreased intratumor proliferation. PT reduced proliferation and apoptosis in wild-type tumors, not in silenced ones. Part of the resistance can be due to the lower cycling of shTFEB cells that make them more protected by the DNA damage elicited by PT. Again, both reduced proliferation and intratumor apoptosis were restored if PT was combined with NZ, in wild-type as well as in shTFEB-silenced tumors. ScRNA-Seq analysis supported these findings: indeed, cancer cell clusters of shTFEB tumors had lower expression of the pro-apoptotic genes *Bax*, *cytochrome c* and *caspase 9*, known to be upregulated by PT [67]. PT did not induce these genes in shTFEB-xenografts, except when it was associated with NZ. This data demonstrate that the NZ + PT combination overcomes TFEB-induced chemoresistance via tumor-intrinsic mechanisms, based on decreased proliferation and increased apoptosis.

Additionally, the efficacy of the combination was also mediated by the enhanced immuno-killing induced by NZ, as confirmed by scRNASEq-based qualitative and quantitative analysis of the immune infiltrate and by the differentially up-regulated immune-related processes. Indeed, shTFEB tumors had a more variegated TIME compared to wild type tumors, but PT treatment turned the TIME of shTFEB tumors toward immunosuppression, by decreasing anti-tumor populations as CD4^+^T-helper lymphocytes, Vγ9Vδ2 T-lymphocytes and NK cells. These results were in line with previous findings, showing that PT promotes a TFEB-dependent up-regulation of HLA-A that in turns increases the immunogenicity of ovarian cancer cells, but at the same time it increases PD-L1 and PD-L2, inducing immune-anergy. This mechanism contributes to the cisplatin resistance in ovarian cancer [68].

TIME composition of shTFEB tumors was completely reshaped by the combination of PT and NZ that increased CD4^+^T-helper lymphocytes and Vγ9Vδ2 T-lymphocytes, as it did in wild-type tumors. In addition, the combination produced an increase in CD8^+^T-cytotoxic lymphocytes and NK cells, coupled with a decrease in Treg cells that were instead up-regulated in wild-type tumors. Overall, the reshaping of TIME induced by NZ is well aligned with the results obtained in ex vivo Vγ9Vδ2 T-cells/NSCLC co-cultures. The expansion of Vγ9Vδ2 T-lymphocytes is of paramount importance in tumor killing: although Vγ9Vδ2 T-cells are only 5% of circulating T-lymphocytes, they can recognize tumor antigens, kill tumor cells through perforin-granzyme B, Fas/FasL and TRAIL pathways [69], and activate CD8^+^T-cytotoxic cells and NK cells [70], amplifying the tumor immuno-killing. These events occur in shTFEB tumors treated with NZ or – to a greater extent – with the combination of NZ + PT, where Vγ9Vδ2 T-cells, CD8^+^T-cytotoxic cells and NK cells were all increased. The comparison between the tumor growth in immunocompetent and immunodeficient mice clarified that: 1) TIME plays a crucial role in determining NSCLC chemoresistance: indeed, the reduction of tumor growth induced by NZ, alone or combined with PT, was higher in immunocompetent than in immunodeficient mice; 2), the combination of NZ + PT was more effective in shTFEB tumors than in wild-type tumor in immunocompetent mice, likely because it reshaped the immuno-suppressive TIME of shTFEB tumors.

The immune-reshaping induced by NZ may counteract the immune-suppressive TIME present in TFEB^low^ABCA1^low^ABCC1^high^ tumors. Indeed, the WGCNA of TCGA-LUAD cohort revealed that patients with this tumor phenotype had down-regulated an extensive gene network led by *SASH3* and including *IKZF1*,* IL10RA*,* CD53* and *SNX20*, all involved in immune activation. *SASH3* is a lymphocytic signal transducer and its deficiency or mutations impairs the development of T-cells, B-cells and NK cells [71]. *IKZF1* is involved in lymphoid differentiation [72] and its co-expression with *SASH3* and *IL10RA* is associated with good prognosis of head and neck squamous cell carcinoma [73]. Also the high expression of *SNX20*, which is connected with *SASH3* and *CD53* through the focal adhesion-encoding gene *LPXN* [74], has been associated with immune-active TIME and better OS in lung adenocarcinoma patients [73]. The downregulation of this immune-related network, consistent with the immune-evasive nature of shTFEB tumors, provides a further explanation for the low survival of TFEB^low^ABCA1^low^ABCC1^high^ NSCLC patients.

## Conclusions

This work unveiled that TFEB is a gatekeeper of the sensitivity to chemotherapy and immuno-killing in NSCLC, because it simultaneously induces ABCA1 and represses ABCC1. We identified the TFEB^low^ABCA1^low^ABCC1^high^ phenotype as predictive of poor response to chemotherapy and immunotherapy in NSCLC patients: the analysis of this gene signature in the diagnostic workflow of NSCLC may give useful indications in choosing the best treatment for each patient. Moreover, by deciphering the molecular and metabolic pathways, and the changes in TIME modulated by TFEB, we identified a novel chemo-immuno-sensitizing strategy, based on the already approved aminobisphosphonate zoledronic acid, for NSCLC. Such strategy was effective in tumors with endogenous detectable levels of TFEB, but also in tumors with reduced levels of TFEB that are more resistant to chemotherapy and more immune-evasive.

### Electronic supplementary material

Below is the link to the electronic supplementary material.

Supplementary Material 1

## Data Availability

All data generated or analyzed during this study are included in this published article and its supplemental information files.

## Abbreviations

NSCLC: non-small cell lung cancer
ABCA1: Adenosine Triphosphate (ATP)-Binding Cassette (ABC) Transporter A1
ABCC1: Adenosine Triphosphate (ATP)-Binding Cassette (ABC) Transporter C1
IPP: Isopentenyl pyrophosphate
TFEB: Transcription factor EB
ERK1/2: Extracellular Signal-regulated Kinase 1/2
mTORC1: Mammalian target of rapamycin complex 1
HIF1a: Hypoxia inducible factor 1 alpha
ChIP-seq: Chromatin immunoprecipitation-sequencing
TCGA: The Cancer Genome Atlas
FBS: Fetal bovine serum
LUAD: Lung adenocarcinoma
VERSUST: variance-stabilized transformed
OS: Overall survival
PFS: Progression free survival
WGCNA: Weighted correlation network analysis
GSVA: Gene set variation analysis
DEG: Differentially expressed genes
BSA: Bovine serum albumin
PBS: Phosphate-saline buffer
PMSF: Phenylmethylsulphonyl fluoride
SREBP2: Sterol regulatory element binding protein 2
GAPDH: Glyceraldehyde 3-phosphate dehydrogenase
DAPI: 4’,6-diamidino-2-phenylindole
TLC: Thin layer chromatography
ETC: Electron transport chain
OXPHOS: Oxidative phosphorylation
OCR: Oxygen consumption rate
NZ: Nano-assembled zoledronic acid
WT: Wild-type
shTFEB: TFEB-silenced
NSG: NOD SCID-γ
s.c.: Subcutaneously
i.v.: Intravenously
RBC: Red blood cells
WBC: white blood cells
Hb: Hemoglobin
PLT: Platelets
LDH: Lactate dehydrogenase
AST: Aspartate aminotransferase
ALT: Alanine aminotransferase
AP: Alkaline phosphatase
CPK: Creatine phosphokinase (CPK)
scRNA-Seq: Single cell RNA-sequencing
CMO: Cell multiplexing oligo
7-AAD: 7-aminoactinomycin D
GEX: Gene expression
PCA: Principal component analysis
UMAP: Uniform manifold approximation and projection
GSEA: Gene set enrichment analysis
ANOVA: One-way analysis of variance
FPPS: Farnesyl pyrophosphate synthase
TIME: Tumor immune micro-environment
TRDC: T cell receptor delta constant
Treg: T-regulatory
NK: Natural killer

## References

[1] Lung cancer statistics | World Cancer Research Fund (WCRF) International. https://www.wcrf.org/cancer-trends/lung-cancer-statistics/. Accessed 19 december 2023.

[2] Franzi S, Mattioni G, Rijavec E, Croci GA, Tosi D. Neoadjuvant Chemo-Immunotherapy for locally Advanced Non-small-cell Lung Cancer: a review of the literature. J Clin Med. 2022;11(9):2629. 35566754 10.3390/jcm11092629 PMC9099888

[3] Gridelli C, Peters S, Mok T, Garassino M, Paz-Ares L, Attili I, et al. Face to face among different chemo-immunotherapy combinations in the first line treatment of patients with advanced non-small cell lung cancer: results of an international expert panel meeting by the Italian association of thoracic oncology (AIOT). Lung Cancer. 2024;187:107441. 38141488 10.1016/j.lungcan.2023.107441

[4] Guo Q, Liu L, Chen Z, Fan Y, Zhou Y, Yuan Z, et al. Current treatments for non-small cell lung cancer. Front Oncol. 2022;12:945102. 36033435 10.3389/fonc.2022.945102 PMC9403713

[5] Sharma P, Hu-Lieskovan S, Wargo JA, Ribas A, Primary. Adaptive and Acquired Resistance to Cancer Immunotherapy. Cell. 2017;168(4):707–23. 28187290 10.1016/j.cell.2017.01.017 PMC5391692

[6] Kachalaki S, Ebrahimi M, Mohamed Khosroshahi L, Mohammadinejad S, Baradaran B. Cancer chemoresistance; biochemical and molecular aspects: a brief overview. Eur J Pharm Sci. 2016;89:20–30. 27094906 10.1016/j.ejps.2016.03.025

[7] Zhang Q, Song Y, Cheng X, Xu Z, Matthew OA, Wang J, et al. Apatinib reverses paclitaxel-resistant Lung Cancer cells (A549) through blocking the function of ABCB1 transporter. Anticancer Res. 2019;39(10):5461–71. 31570440 10.21873/anticanres.13739

[8] Robey RW, Pluchino KM, Hall MD, Fojo AT, Bates SE, Gottesman MM. Revisiting the role of ABC transporters in multidrug-resistant cancer. Nat Rev Cancer. 2018;18(7):452–64. 29643473 10.1038/s41568-018-0005-8 PMC6622180

[9] Castella B, Kopecka J, Sciancalepore P, Mandili G, Foglietta M, Mitro N, et al. The ATP-binding cassette transporter A1 regulates phosphoantigen release and Vγ9Vδ2 T cell activation by dendritic cells. Nat Commun. 2017;8(1):15663. 28580927 10.1038/ncomms15663 PMC5465356

[10] Salaroglio IC, Belisario DC, Akman M, La Vecchia S, Godel M, Anobile DP, et al. Mitochondrial ROS drive resistance to chemotherapy and immune-killing in hypoxic non-small cell lung cancer. J Exp Clin Cancer Res. 2022;41(1):243. 35953814 10.1186/s13046-022-02447-6 PMC9373288

[11] Belisario DC, Akman M, Godel M, Campani V, Patrizio MP, Scotti L, et al. ABCA1/ABCB1 ratio determines chemo- and Immune-Sensitivity in Human Osteosarcoma. Cells. 2020;9(3):647. 32155954 10.3390/cells9030647 PMC7140509

[12] Alexa-Stratulat T, Pešić M, Gašparović AČ, Trougakos IP, Riganti C. What sustains the multidrug resistance phenotype beyond ABC efflux transporters? Looking beyond the tip of the iceberg. Drug Resist Updat. 2019;46:100643. 31493711 10.1016/j.drup.2019.100643

[13] Gonçalves AC, Richiardone E, Jorge J, Polónia B, Xavier CPR, Salaroglio IC, et al. Impact of cancer metabolism on therapy resistance - clinical implications. Drug Resist Updat. 2021;59:100797. 34955385 10.1016/j.drup.2021.100797

[14] Salaroglio IC, Belisario DC, Bironzo P, Ananthanarayanan P, Ricci L, Digiovanni S, et al. SKP2 drives the sensitivity to neddylation inhibitors and cisplatin in malignant pleural mesothelioma. J Exp Clin Cancer Res. 2022;41(1):75. 35197103 10.1186/s13046-022-02284-7 PMC8864928

[15] Hussein NA, Malla S, Pasternak MA, Terrero D, Brown NG, Ashby CR, et al. The role of endolysosomal trafficking in anticancer drug resistance. Drug Resist Updat. 2021;57:100769. 34217999 10.1016/j.drup.2021.100769

[16] Settembre C, Di Malta C, Polito VA, Garcia Arencibia M, Vetrini F, Erdin S, et al. TFEB links autophagy to lysosomal biogenesis. Science. 2011;332(6036):1429–33. 21617040 10.1126/science.1204592 PMC3638014

[17] Puertollano R, Ferguson SM, Brugarolas J, Ballabio A. The complex relationship between TFEB transcription factor phosphorylation and subcellular localization. EMBO J. 2018;37(11):98804. 10.15252/embj.201798804 PMC5983138 29764979

[18] Settembre C, Ballabio A. Lysosome: regulator of lipid degradation pathways. Trends Cell Biol. 2014;24(12):743–50. 25061009 10.1016/j.tcb.2014.06.006 PMC4247383

[19] Li M, Wang Z, Wang P, Li H, Yang L. TFEB: A Emerging Regulator in lipid homeostasis for atherosclerosis. Front Physiol. 2021;12:63992. 10.3389/fphys.2021.639920 PMC7925399 33679452

[20] Li Y, Hodge J, Liu Q, Wang J, Wang Y, Evans TD, et al. TFEB is a master regulator of tumor-associated macrophages in breast cancer. J Immunother Cancer. 2020;8(1):e000543. 32487570 10.1136/jitc-2020-000543 PMC7269543

[21] Doronzo G, Astanina E, Corà D, Chiabotto G, Comunanza V, Noghero A, et al. TFEB controls vascular development by regulating the proliferation of endothelial cells. EMBO J. 2019;38(3):e98250. 30591554 10.15252/embj.201798250 PMC6356157

[22] Vesel M, Rapp J, Feller D, Kiss E, Jaromi L, Meggyes M, et al. ABCB1 and ABCG2 drug transporters are differentially expressed in non-small cell lung cancers (NSCLC) and expression is modified by cisplatin treatment via altered wnt signaling. Respir Res. 2017;18(1):52. 28340578 10.1186/s12931-017-0537-6 PMC5364604

[23] Amoêdo ND, Castelo-Branco MTL, Paschoal MEM, Pezzuto P, Esperança ABT, Rumjanek VM, et al. Expression of ABC transporters, p53, Bax, Bcl-2 in an archival sample of non-small cell lung cancer bearing a deletion in the EGFR gene. Int J Mol Med. 2009;23(5):609–14. 19360319 10.3892/ijmm_00000171

[24] https://portal.gdc.cancer.gov/projects/TCGA-LUAD. Accessed on 20 September 2022.

[25] Carlson M, Bioconductor. 2019. http://bioconductor.org/packages/org.Hs.eg.db/. Accessed: 16 Dec 2022.

[26] Love MI, Huber W, Anders S. Moderated estimation of Fold change and dispersion for RNA-seq data with DESeq2. Genome Biol. 2014;15(12):550. 25516281 10.1186/s13059-014-0550-8 PMC4302049

[27] Langfelder P, Horvath S. WGCNA: an R package for weighted correlation network analysis. BMC Bioinformatics. 2008;9(1):559. 19114008 10.1186/1471-2105-9-559 PMC2631488

[28] Ariano C, Costanza F, Akman M, Riganti C, Corà D, Casanova E, et al. TFEB inhibition induces melanoma shut-down by blocking the cell cycle and rewiring metabolism. Cell Death Dis. 2023;14(5):314. 37160873 10.1038/s41419-023-05828-7 PMC10170071

[29] https://jaspar. genereg.net/. Accessed on 14 January 2023.

[30] Castella B, Riganti C, Fiore F, Pantaleoni F, Canepari ME, Peola S, et al. Immune modulation by zoledronic acid in human myeloma: an advantageous cross-talk between Vγ9Vδ2 T cells, αβ CD8 + T cells, regulatory T cells, and dendritic cells. J Immunol. 2011;187(4):1578–90. 21753152 10.4049/jimmunol.1002514

[31] Teixeira RG, Belisario DC, Fontrodona X, Romero I, Tomaz AI, Garcia MH, et al. Unprecedented collateral sensitivity for cisplatin-resistant lung cancer cells presented by new ruthenium organometallic compounds. Inorg Chem Front. 2021;8(8):1983–96. 10.1039/D0QI01344G

[32] Kopecka J, Porto S, Lusa S, Gazzano E, Salzano G, Pinzòn-Daza ML, et al. Zoledronic acid-encapsulating self-assembling nanoparticles and doxorubicin: a combinatorial approach to overcome simultaneously chemoresistance and immunoresistance in breast tumors. Oncotarget. 2016;7(15):20753–72. 26980746 10.18632/oncotarget.8012 PMC4991490

[33] Salaroglio IC, Gazzano E, Abdullrahman A, Mungo E, Castella B, Abd-Elrahman GEFA, et al. Increasing intratumor C/EBP-β LIP and nitric oxide levels overcome resistance to doxorubicin in triple negative breast cancer. J Exp Clin Cancer Res. 2018;37(1):286. 30482226 10.1186/s13046-018-0967-0 PMC6258159

[34] Barkas N, Petukhov V, Nikolaeva D, Lozinsky Y, Demharter S, Khodosevich K, et al. Joint analysis of heterogeneous single-cell RNA-seq dataset collections. Nat Methods. 2019;16(8):695–8. 31308548 10.1038/s41592-019-0466-z PMC6684315

[35] Hao Y, Stuart T, Kowalski MH, Choudhary S, Hoffman P, Hartman A, et al. Dictionary learning for integrative, multimodal and scalable single-cell analysis. Nat Biotechnol. 2024;42(2):293–304. 37231261 10.1038/s41587-023-01767-y PMC10928517

[36] Stuart T, Butler A, Hoffman P, Hafemeister C, Papalexi E, Mauck WM, et al. Comprehensive Integration of Single-Cell Data. Cell. 2019;177(7):1888–902. 31178118 10.1016/j.cell.2019.05.031 PMC6687398

[37] Barkas N, Petukhov V, Kharchenko P, Steiger S, Rydbirk R. Evan Biederstedt. Pagoda2: Single Cell Analysis and Differential Expression. R package version 1.0.12 (2021).

[38] http://pklab.med.harvard.edu/peterk/p2/smallMem2.local/index.html. Accessed on 24 June 2024.

[39] Yu G, Wang LG, Han Y, He QY. clusterProfiler: an R package for comparing biological themes among gene clusters. OMICS. 2012;16(5):284–7. 22455463 10.1089/omi.2011.0118 PMC3339379

[40] https://geneontology.org/. Accessed on 15 July 2024.

[41] Czuba LC, Hillgren KM, Swaan PW. Post-translational modifications of transporters. Pharmacol Ther. 2018;192:88–99. 29966598 10.1016/j.pharmthera.2018.06.013 PMC6263853

[42] Kopecka J, Godel M, Dei S, Giampietro R, Belisario DC, Akman M, et al. Insights into P-Glycoprotein inhibitors: New inducers of Immunogenic Cell Death. Cells. 2020;9(4):1033. 32331368 10.3390/cells9041033 PMC7226521

[43] Petruzzelli R, Mariniello M, De Cegli R, Catalano F, Guida F, Di Schiavi E, et al. TFEB regulates ATP7B expression to Promote Platinum Chemoresistance in Human Ovarian Cancer cells. Cells. 2022;11(2):219. 35053335 10.3390/cells11020219 PMC8774088

[44] Arito M, Horiba T, Hachimura S, Inoue J, Sato R. Growth factor-induced phosphorylation of sterol regulatory element-binding proteins inhibits sumoylation, thereby stimulating the expression of their target genes, low density lipoprotein uptake, and lipid synthesis. J Biol Chem. 2008;283(22):15224–31. 18403372 10.1074/jbc.M800910200 PMC3258893

[45] Monteiro JP, Oliveira PJ, Jurado AS. Mitochondrial membrane lipid remodeling in pathophysiology: a new target for diet and therapeutic interventions. Prog Lipid Res. 2013;52(4):513–28. 23827885 10.1016/j.plipres.2013.06.002

[46] Calabrese C, Nolte H, Pitman MR, Ganesan R, Lampe P, Laboy R, et al. Mitochondrial translocation of TFEB regulates complex I and inflammation. EMBO Rep. 2024;25(2):704–24. 38263327 10.1038/s44319-024-00058-0 PMC10897448

[47] Giddings EL, Champagne DP, Wu MH, Laffin JM, Thornton TM, Valenca-Pereira F, et al. Mitochondrial ATP fuels ABC transporter-mediated drug efflux in cancer chemoresistance. Nat Commun. 2021;12:2804. 33990571 10.1038/s41467-021-23071-6 PMC8121950

[48] Haque A, Baig GA, Alshawli AS, Sait KHW, Hafeez BB, Tripathi MK, et al. Interaction Analysis of MRP1 with anticancer drugs used in ovarian Cancer: in Silico Approach. Life. 2022;12(3):383. 35330134 10.3390/life12030383 PMC8954655

[49] Zeng W, Zheng S, Mao Y, Wang S, Zhong Y, Cao W, et al. Elevated N-Glycosylation contributes to the Cisplatin Resistance of Non-small Cell Lung Cancer cells revealed by membrane proteomic and Glycoproteomic Analysis. Front Pharmacol. 2021;12:805499. 35002739 10.3389/fphar.2021.805499 PMC8728018

[50] Räikkönen J, Taskinen M, Dunford JE, Mönkkönen H, Auriola S, Mönkkönen J. Correlation between time-dependent inhibition of human farnesyl pyrophosphate synthase and blockade of mevalonate pathway by nitrogen-containing bisphosphonates in cultured cells. Biochem Biophys Res Commun. 2011;407(4):663–7. 21420384 10.1016/j.bbrc.2011.03.070

[51] Ristori S, Grillo I, Lusa S, Thamm J, Valentino G, Campani V, et al. Structural characterization of self-assembling hybrid nanoparticles for bisphosphonate delivery in tumors. Mol Pharm. 2018;15(3):1258–65. 29433321 10.1021/acs.molpharmaceut.7b01085

[52] Halkias J, Yen B, Taylor KT, Reinhartz O, Winoto A, Robey EA, et al. Conserved and divergent aspects of human T-cell development and migration in humanized mice. Immunol Cell Biol. 2015;93(8):716–26. 25744551 10.1038/icb.2015.38 PMC4575952

[53] Petersen B, Kammerer R, Frenzel A, Hassel P, Dau TH, Becker R, et al. Generation and first characterization of TRDC-knockout pigs lacking γδ T cells. Sci Rep. 2021;11:14965. 34294758 10.1038/s41598-021-94017-7 PMC8298467

[54] Mohammad IS, He W, Yin L. Understanding of human ATP binding cassette superfamily and novel multidrug resistance modulators to overcome MDR. Biomed Pharmacother. 2018;100:335–48. 29453043 10.1016/j.biopha.2018.02.038

[55] Wang Y, Wang Y, Qin Z, Cai S, Yu L, Hu H, et al. The role of non-coding RNAs in ABC transporters regulation and their clinical implications of multidrug resistance in cancer. Expert Opin Drug Metab Toxicol. 2021;17(3):291–306. 33544643 10.1080/17425255.2021.1887139

[56] Giatromanolaki A, Kalamida D, Sivridis E, Karagounis IV, Gatter KC, Harris AL, et al. Increased expression of transcription factor EB (TFEB) is associated with autophagy, migratory phenotype and poor prognosis in non-small cell lung cancer. Lung Cancer. 2015;90(1):98–105. 26264650 10.1016/j.lungcan.2015.07.008

[57] Munoz M, Henderson M, Haber M, Norris M. Role of the MRP1/ABCC1 multidrug transporter protein in cancer. IUBMB Life. 2007;59(12):752–7. 18085475 10.1080/15216540701736285

[58] Fang L, Sheng H, Wan D, Zhu C, Jiang R, Sun X, et al. Prognostic role of multidrug resistance-associated protein 1 expression and platelet count in operable non-small cell lung cancer. Oncol Lett. 2018;16(1):1123–32. 30061938 10.3892/ol.2018.8763 PMC6063026

[59] Liu K, Zhang W, Tan J, Ma J, Zhao J. MiR-200b-3p functions as an Oncogene by Targeting ABCA1 in Lung Adenocarcinoma. Technol Cancer Res Treat. 2019;18:1533033819892590. 31795847 10.1177/1533033819892590 PMC6893970

[60] Zhitomirsky B, Assaraf YG. Lysosomes as mediators of drug resistance in cancer. Drug Resist Updat. 2016;24:23–33. 26830313 10.1016/j.drup.2015.11.004

[61] Chu HY, Wang W, Chen X, Jiang YE, Cheng R, Qi X, et al. Bafilomycin A1 increases the sensitivity of tongue squamous cell carcinoma cells to cisplatin by inhibiting the lysosomal uptake of platinum ions but not autophagy. Cancer Lett. 2018;423:105–12. 29524554 10.1016/j.canlet.2018.03.003

[62] Appelqvist H, Wäster P, Kågedal K, Öllinger K. The lysosome: from waste bag to potential therapeutic target. J Mol Cell Biol. 2013;5(4):214–26. 23918283 10.1093/jmcb/mjt022

[63] Settembre C, De Cegli R, Mansueto G, Saha PK, Vetrini F, Visvikis O, et al. TFEB controls cellular lipid metabolism through a starvation-induced autoregulatory loop. Nat Cell Biol. 2013;15(6):647–58. 23604321 10.1038/ncb2718 PMC3699877

[64] Li X, Zhang X, Zheng L, Kou J, Zhong Z, Jiang Y, et al. Hypericin-mediated sonodynamic therapy induces autophagy and decreases lipids in THP-1 macrophage by promoting ROS-dependent nuclear translocation of TFEB. Cell Death Dis. 2016;7(12):e2527. 28005078 10.1038/cddis.2016.433 PMC5260986

[65] Goicoechea L, de la Conde L, Torres S, García-Ruiz C, Fernández-Checa JC. Mitochondrial cholesterol: metabolism and impact on redox biology and disease. Redox Biol. 2023;61:102643. 36857930 10.1016/j.redox.2023.102643 PMC9989693

[66] Li Y, Shi L, Zhao F, Luo Y, Zhang M, Wu X, et al. PIM1 attenuates cisplatin-induced AKI by inhibiting Drp1 activation. Cell Signal. 2024;113:110969. 37967691 10.1016/j.cellsig.2023.110969

[67] Shan J, Kimura H, Yokoi S, Kamiyama K, Imamoto T, Takeda I, et al. PPAR-δ activation reduces cisplatin-induced apoptosis via inhibiting p53/Bax/caspase-3 pathway without modulating autophagy in murine renal proximal tubular cells. Clin Exp Nephrol. 2021;25(6):598–607. 33646450 10.1007/s10157-021-02039-2

[68] Liu W, Wang Y, Xie Y, Dai T, Fan M, Li C, et al. Cisplatin remodels the tumor immune microenvironment via the transcription factor EB in ovarian cancer. Cell Death Discov. 2021;7(1):136. 34091590 10.1038/s41420-021-00519-8 PMC8179924

[69] Li Y, Li G, Zhang J, Wu X, Chen X. The dual roles of human γδ T Cells: Anti-tumor or Tumor-promoting. Front Immunol. 2021;11:619954. 33664732 10.3389/fimmu.2020.619954 PMC7921733

[70] Lee D, Rosenthal CJ, Penn NE, Dunn ZS, Zhou Y, Yang L. Human γδ T cell subsets and their clinical applications for Cancer Immunotherapy. Cancers. 2022;14(12):3005. 35740670 10.3390/cancers14123005 PMC9221220

[71] Delmonte OM, Bergerson JRE, Kawai T, Kuehn HS, McDermott DH, Cortese I, et al. SASH3 variants cause a novel form of X-linked combined immunodeficiency with immune dysregulation. Blood. 2021;138(12):1019–33. 33876203 10.1182/blood.2020008629 PMC8462359

[72] Yang L, Luo Y, Wei J. Integrative genomic analyses on Ikaros and its expression related to solid cancer prognosis. Oncol Rep. 2010;24(2):571–7. 20596648 10.3892/or_00000894

[73] Wang J, Tian Y, Zhu G, Li Z, Wu Z, Wei G, et al. Establishment and validation of immune microenvironmental gene signatures for predicting prognosis in patients with head and neck squamous cell carcinoma. Int Immunopharmacol. 2021;97:107817. 34091115 10.1016/j.intimp.2021.107817

[74] Tanaka T, Moriwaki K, Murata S, Miyasaka M. LIM domain-containing adaptor, leupaxin, localizes in focal adhesion and suppresses the integrin-induced tyrosine phosphorylation of paxillin. Cancer Sci. 2010;101(2):363–8. 19917054 10.1111/j.1349-7006.2009.01398.x PMC11158308

