# Correction: TFEB controls sensitivity to chemotherapy and immuno-killing in non-small cell lung cancer

**DOI:** 10.1186/s13046-024-03170-0

**Published:** 2024-08-29

**Authors:** Muhlis Akman, Ciro Monteleone, Gabriella Doronzo, Martina Godel, Francesca Napoli, Alessandra Merlini, Virginia Campani, Valeria Nele, Elisa Balmas, Tatiana Chontorotzea, Simona Fontana, Sabrina Digiovanni, Francesca Alice Barbu, Elena Astanina, Niloufar Jafari, Iris Chiara Salaroglio, Joanna Kopecka, Giuseppe De Rosa, Thomas Mohr, Alessandro Bertero, Luisella Righi, Silvia Novello, Giorgio Vittorio Scagliotti, Federico Bussolino, Chiara Riganti

**Affiliations:** 1 Department of Oncology, University of Torino, Torino, Italy; 2 Candiolo Cancer Institute, FPO-IRCCS, Candiolo, Turin, Italy; 3 Pathology Unit, Department of Oncology at San Luigi Hospital, University of Torino, Torino, Italy; 4 Thoracic Oncology Unit, Department of Oncology at San Luigi Hospital, University of Torino, Torino, Italy; 5 Department of Pharmacy, University of Napoli Federico II, Napoli, Italy; 6 Department of Molecular Biotechnology and Health Sciences, University of Torino, Torino, Italy; 7 Center for Cancer Research and Comprehensive Cancer Center, Medical University of Vienna, Vienna, Austria; 8 Molecular Biotechnology Center “Guido Tarone”, University of Torino, Torino, Italy

**Correction: J Exp Clin Cancer Res 43**,** 219 (2024)**

**https://doi.org/10.1186/s13046-024-03142-4**

Following publication of the original article [1], the authors found out errors pertaining on the affiliations of the authors. The changes requested are highlighted in bold.

• Affiliation 2 should be changed from: “IRCCS Candiolo Cancer Institute, Candiolo, Italy” to “**Candiolo Cancer Institute**,** FPO-IRCCS**,** Candiolo**,** Turin**,** Italy**”.

• Affiliation 5 should be updated from: “Department of Pharmacy, University of Napoli “Federico II”, Napoli, Italy” to “Department of Pharmacy, University of Napoli **Federico II**, Napoli, Italy”.

• Affiliation 7 should be updated from: “Medical University of Vienna, Vienna, Austria” to “**Center for Cancer Research and Comprehensive Cancer Center**, Medical University of Vienna, Vienna, Austria”.

These corrections do not affect the overall result or conclusion of the article. The original article [1] has been corrected.

## References

[1] Akman M, Monteleone C, Doronzo G, et al. TFEB controls sensitivity to chemotherapy and immuno-killing in non-small cell lung cancer. J Exp Clin Cancer Res. 2024;43:219. 10.1186/s13046-024-03142-4. 39107857 10.1186/s13046-024-03142-4 PMC11304671

