# Supplementary material for: TFEB controls sensitivity to chemotherapy and immuno-killing in non-small cell lung cancer
Source: J Exp Clin Cancer Res. 2024 Aug 7;43:219. doi: 10.1186/s13046-024-03142-4 (PMC11304671; doi:10.1186/s13046-024-03142-4)
Supplement: Supplementary file 1 — Supplementary Material 1 [file 13046_2024_3142_MOESM1_ESM.pdf]

SUPPLEMENTAL DATA

Supplemental Table S1. Demographic features of TCGA-LUAD cohort patients

TCGA-LUAD Dataset (n = 585)	Male	Female
Patients with Primary Tumor (n=531)	246	287
Patients with Normal Tissue (n=59)	25	34

Supplemental Table S2. Clinical follow-up and gene expression data in the retrospective cohort of patients analyzed at the Department of Oncology, University of Torino, Italy

Patient code	1st line treatment	PFS (months)	OS (months)	TFEB (relative expression)	ABCC1 (relative expression)	ABCA1 (relative expression)
**16-C-00249**	ICI-based immunotherapy	18	21	2.13	0.52	2.29
**17-C-007826**	ICI-based immunotherapy	24	26	2.56	0.41	3.29
**17-C-04337**	ICI-based immunotherapy	8	5	0.15	2.96	0.21
**17-C-04697**	ICI-based immunotherapy	12	21	2.58	0.61	1.98
**17-C-04914 B**	ICI-based immunotherapy	23	26	3.51	0.42	3.15
**17-C-05257 A**	ICI-based immunotherapy	23	30	3.69	0.28	4.05
**17-I-00793A2**	ICI-based immunotherapy	21	28	3.01	1.04	3.24
**17-I-10155**	ICI-based immunotherapy	20	24	2.14	0.62	2.56
**17-I-10421**	ICI-based immunotherapy	37	46	4.01	0.14	4.5
**17-I-12097 B1**	ICI-based immunotherapy	35	54	6.12	0.29	4.11
**17-I-12406**	ICI-based immunotherapy	21	26	2.95	0.61	3.59
**17-I-12748**	ICI-based immunotherapy	23	23	2.54	0.42	2.93
**17-I-13622A2**	ICI-based immunotherapy	20	26	3.02	0.69	3.17
**17-I-13953 A**	ICI-based immunotherapy	18	28	2.04	0.46	2.51
**17-I-14050**	ICI-based immunotherapy	27	38	3.24	0.84	3.54

**18-C-00265**	ICI-based immunotherapy	9	19	1.47	0.54	1.97
**18-C-01105 B**	ICI-based immunotherapy	22	29	2.08	0.59	2.11
**18-C-04462**	ICI-based immunotherapy	41	53	3.96	0.04	4.96
**18-C-04699**	ICI-based immunotherapy	8	15	0.42	1.56	0.51
**18-C-05582 B**	ICI-based immunotherapy	58	71	4.92	0.14	5.36
**18-I-00097 B**	ICI-based immunotherapy	24	31	2.11	0.39	2.58
**18-I-01832**	ICI-based immunotherapy	22	27	2.56	0.42	2.14
**18-I-02470A2**	ICI-based immunotherapy	51	59	4.85	0.18	5.46
**18-I-05255**	ICI-based immunotherapy	29	50	3.28	0.28	4.02
**18-I-05700A2**	ICI-based immunotherapy	6	20	1.14	0.14	1.47
**18-I-06387 B2**	ICI-based immunotherapy	3	9	0.15	2.15	0.26
**18-I-07856**	ICI-based immunotherapy	14	22	0.81	0.97	1.36
**18-I-10670**	ICI-based immunotherapy	23	28	0.96	1.13	1.82
**18-I-11188**	ICI-based immunotherapy	24	45	1.25	0.42	1.98
**19-C-01646**	ICI-based immunotherapy	35	49	2.28	0.21	2.45
**19-C-5330**	ICI-based immunotherapy	15	55	3.15	0.17	3.85
**19-I-01684**	ICI-based immunotherapy	21	30	2.84	0.52	3.24
**19-I-08588**	ICI-based immunotherapy	4	5	0.08	2.36	0.12

**19-I-5272**	ICI-based immunotherapy	1	2	0.45	3.18	0.39
**20-C-02138**	ICI-based immunotherapy	11	23	1.91	1.15	2.15
**20-C-2023**	ICI-based immunotherapy	32	46	3.15	0.18	3.58
**20-I-00099**	ICI-based immunotherapy	2	5	0.08	2.89	0.14
**20-I-00644 B1**	ICI-based immunotherapy	29	63	4.16	0.09	4.28
**20-I-04286**	ICI-based immunotherapy	9	19	1.28	1.47	1.58
**20-I-2504**	ICI-based immunotherapy	9	11	1.11	1.63	1.23
**20-I-4408**	ICI-based immunotherapy	43	53	4.52	0.05	4.65
**ITACA 135**	Pt-based chemotherapy	5	11	0.81	1.51	1.12
**ITACA 15**	Pt-based chemotherapy	45	63	5.11	0.17	5.64
**ITACA 157**	Pt-based chemotherapy	4	14	0.76	1.28	0.85
**ITACA 205**	Pt-based chemotherapy	1	7	0.38	2.41	0.25
**ITACA 206**	Pt-based chemotherapy	4	11	0.94	1.48	0.62
**ITACA 24**	Pt-based chemotherapy	6	15	0.56	1.52	0.78
**ITACA 242**	Pt-based chemotherapy	15	16	0.81	1.34	0.72
**ITACA 244**	Pt-based chemotherapy	2	19	1.17	0.91	1.58
**ITACA 292**	Pt-based chemotherapy	3	5	0.29	2.25	0.09
**ITACA 348**	Pt-based chemotherapy	14	19	2.15	0.81	1.85

**ITACA 383**	Pt-based chemotherapy	1	2	0.17	3.26	0.23
**ITACA 426**	Pt-based chemotherapy	1	3	0.26	3.82	0.07
**ITACA 446**	Pt-based chemotherapy	1	6	0.11	2.81	0.19
**ITACA 544**	Pt-based chemotherapy	2	3	0.18	3.45	0.08
**ITACA 587**	Pt-based chemotherapy	29	45	3.69	0.15	4.28
**ITACA 607**	Pt-based chemotherapy	1	2	0.48	4.11	0.19
**ITACA 623**	Pt-based chemotherapy	12	30	2.54	1.25	3.15
**ITACA 624**	Pt-based chemotherapy	1	3	0.23	0.46	0.42
**ITACA 631**	Pt-based chemotherapy	2	5	0.28	0.28	0.53
**ITACA 641**	Pt-based chemotherapy	29	41	3.46	0.21	3.78
**ITACA 670**	Pt-based chemotherapy	8	23	2.07	1.14	2.14
**ITACA 671**	Pt-based chemotherapy	2	6	0.31	2.58	0.32
**ITACA 690**	Pt-based chemotherapy	3	6	0.46	2.46	0.25
**ITACA 715**	Pt-based chemotherapy	47	130	5.28	0.09	6.12
**ITACA 745**	Pt-based chemotherapy	3	11	0.72	1.34	0.81
**ITACA 757**	Pt-based chemotherapy	30	33	2.16	1.22	2.98
**ITACA 759**	Pt-based chemotherapy	27	40	3.59	0.32	4.15
**ITACA 87**	Pt-based chemotherapy	1	6	0.14	2.15	0.22

**ITACA166**	Pt-based chemotherapy	22	32	2.19	0.76	2.76
**ITACA301**	Pt-based chemotherapy	15	28	2.04	0.92	2.15
**ITACA636**	Pt-based chemotherapy	14	16	1.92	0.72	1.15
**MOLEC. 1**	ICI-based immunotherapy	2	4	0.41	3.15	0.36
**MOLEC. 2**	ICI-based immunotherapy	9	17	1.12	1.08	1.24
**SI ITACA**	Pt-based chemotherapy	21	33	2.73	0.78	2.84

Pt: cisplatin or carboplatin; ICI: immune checkpoint inhibitors; PFS: progression free survival; OS: overall survival. *TFEB*, *ABCA1* and *ABCC1* levels were expressed as relative expression toward the housekeeping gene *B2M*.

Supplemental Table S3. Primer sequences used in qRT-PCR and ChIP

**Primers**	**Forward**	**Reverse**
TFEB	5'-GACTCAGAAGCGAGAGCTAACA	5'-TGTGATTGTCTTTCTTCTGCCG
ABCA1	5'-CAGAGCTCACAGCAGGGAC	5'-CTTCTCCGGAAGGCTTGTC
ABCC1	5' -TCTGGTCAGCCCAACTCTCT	5'-CCTGTGATCCACCAGAAGGT
ATP7B	5’-TCTCTGGTCATCCTGGTGGTT-3’	5’-GGGCTTCTGAGGTTTTGCTCT-3’
B2M	5' -AGCAAGGACTGGTCTTTCTATCTC	5'-ATOTCTCCATCCCACTTAAGTATCTT
**TFEB sites on the promoter of**		
ABCA1	GGACCCTAAGACACCTGCTG	TTCCCGGCCTCTGTTTATGT
ABCC1	ACCTCAGTTTCCCCATCTGT	AAGAAACCCAGGTGCAGAGA

Supplemental Table S4. Cholesterol homeostasis-related genes analyzed by PCR Array in wild-type and TFEB-silenced cells

NCI-H2228 cells:

Target	WT Normalized Expression	shTFEB Normalized Expression	shTFEB fold change	Compared to Regulation Threshold
ABCA1	0.00632	0.00552	-1.14395	No change
ABCG1	0.0382	0.01617	-2.36338	Down-regulated*
ACAA2	0.04657	0.01143	-4.07516	Down-regulated**
ACTB	5.19942	2.66482	-1.95114	Down-regulated*
ANKRA2	0.05191	0.0452	-1.14843	No change
APOD	0.01544	0.01036	-1.48958	No change
APOE	0.00049	0.00008	-5.81657	Down-regulated**
APOL1	0.02616	0.01122	-2.33151	Down-regulated*
APOL2	0.028	0.00999	-2.80211	Down-regulated*
CNBP	0.27238	0.26009	-1.04726	No change
CXCL16	0.05064	0.03065	-1.65227	Down-regulated*
CYB5R3	0.06758	0.01504	-4.49319	Down-regulated*
CYP39A1	0.00102	0.00124	1.21801	No change
CYP51A1	0.03726	0.06578	1.76542	Up-regulated*
DHCR24	0.0476	0.01658	-2.87171	Down-regulated*
DHCR7	0.0146	0.0033	-4.42772	Down-regulated**
FDFT1	0.0315	0.00065	-48.40565	Down-regulated***
FDPS	0.38113	0.20606	-1.84955	Down-regulated*
GUSB	0.22555	0.11849	-1.90363	Down-regulated*
HDLBP	0.45042	0.28273	-1.59308	Down-regulated*
HMGCR	0.00263	0.00177	-1.48091	No change
HMGCS1	0.00454	0.01664	3.66876	Up-regulated**
IDI1	0.04883	0.0637	1.30451	No change
INSIG1	0.09391	0.0647	-1.45142	No change
INSIG2	0.02382	0.04048	1.69928	Up-regulated*
LCAT	0.00172	0.00024	-7.26568	Down-regulated**
LDLR	0.03474	0.03437	-1.0107	No change
LDLRAP1	0.01569	0.0065	-2.41377	Down-regulated*

LIPE	0.00531	0.0026	-2.04656	Down-regulated*
LRP10	0.02839	0.01523	-1.86392	Down-regulated*
LRP12	0.01871	0.01645	-1.13739	No change
LRP6	0.01205	0.01351	1.12065	No change
LRPAP1	0.0705	0.04844	-1.45529	No change
MBTPS1	0.11263	0.04245	-2.65347	Down-regulated*
MVD	0.00397	0.00098	-4.03232	Down-regulated**
NR1H4	0.00464	0.00129	-3.59611	Down-regulated**
NSDHL	0.06324	0.04451	-1.42061	No change
OSBPL1A	0.00946	0.01184	1.25146	No change
OSBPL5	0.00508	0.00028	-17.95912	Down-regulated***
PCSK9	0.002	0.00113	-1.76333	Down-regulated*
PMVK	0.05994	0.03129	-1.91548	Down-regulated*
PPARD	0.01303	0.00343	-3.7973	Down-regulated**
PRKAA2	0.00265	0.00639	2.40644	Up-regulated**
PRKAG2	0.06931	0.05961	-1.16277	No change
RPLP0	2.69508	2.21951	-1.21427	No change
SCAP	0.02359	0.00826	-2.85598	Down-regulated**
SCARF1	0.00003	0.0003	10.36432	Up-regulated***
SNX17	0.22834	0.07601	-3.00394	Down-regulated**
SOAT1	0.02597	0.00666	-3.90041	Down-regulated**
SORL1	0.01369	0.00885	-1.54709	No change
SREBF1	0.02128	0.01043	-2.04095	Down-regulated*
SREBF2	0.01569	0.00878	-1.78666	Down-regulated*
STARD3	0.02747	0.01865	-1.47259	No change
TM7SF2	0.29261	0.22981	-1.27327	No change
TRERF1	0.00745	0.00321	-2.32198	Down-regulated*
VLDLR	0.01075	0.0071	-1.51412	No change

NCI-H441 cells

**Target**	**WT Normalized Expression**	**shTFEB Normalized Expression**	**shTFEB fold change**	**Compared to Regulation Threshold**
ABCA1	0.01331	0.0287	2.15663	Up-regulated*
ABCG1	0.04606	0.00103	-44.60911	Down-regulated***
ACAA2	0.00234	0.00387	1.65344	No change

ACTB	2.4819	1.88008	-1.3201	No change
AKR1D1	0.0028	0.00203	-1.3773	No change
ANKRA2	0.15805	0.13815	-1.14404	No change
APOA2	0.00593	0.00009	-65.0202	Down-regulated***
APOA4	0.00015	0.0003	2.02819	Up-regulated*
APOD	0.00084	0.00071	-1.18445	No change
APOE	0.00027	0.00011	-2.43598	Down-regulated**
APOF	0.00133	0.00002	-81.02505	Down regulated***
APOL1	0.03516	0.01608	-2.18581	Down-regulated**
APOL2	0.01202	0.00813	-1.47862	No change
CDH13	0.00431	0.00096	-4.48992	Down-regulated**
CELA3B	0.00005	0.00002	-2.2615	Down-regulated**
CNBP	2.09116	2.49292	1.19212	No change
CXCL16	0.02099	0.03593	1.71165	Up-regulated*
CYB5R3	0.037	0.02927	-1.26422	No change
CYP11A1	0.00121	0.00037	-3.27162	Down-regulated**
CYP39A1	0.00335	0.00381	1.13462	No change
CYP51A1	0.19914	0.27185	1.36516	No change
CYP7B1	0.00626	0.00777	1.24155	No change
DHCR24	0.16111	0.17103	1.0616	No change
DHCR7	0.17051	0.13457	-1.26706	No change
FDFT1	0.07277	0.20263	2.78455	Up-regulated**
FDPS	0.83331	0.53098	-1.56938	No change
GUSB	0.21435	0.15087	-1.42079	No change
HDLBP	0.85871	0.63182	-1.35911	No change
HMGCR	0.04428	0.066	1.49064	No change
HMGCS1	0.02989	0.18492	6.18569	Up-regulated**
IDI1	0.38176	0.30556	-1.24936	No change
INSIG1	0.5445	0.46502	-1.17092	No change
INSIG2	0.49204	0.29921	-1.64447	No change
LCAT	0.03399	0.00167	-20.40966	Down-regulated***
LDLR	0.16837	0.22662	1.34596	No change
LDLRAP1	0.02131	0.00986	-2.16103	Down-regulated**
LIPE	0.00122	0.00104	-1.1796	No change
LRP10	0.04788	0.02033	-2.35542	Down-regulated**

LRP12	0.11268	0.18731	1.66228	Up-regulated*
LRP6	0.08824	0.13293	1.50646	No change
LRPAP1	0.12919	0.14528	1.12456	No change
MBTPS1	0.25246	0.48357	1.91545	Up-regulated*
MVD	0.0066	0.00492	-1.34042	No change
MVK	0.28939	0.07591	-3.81244	Down-regulated**
NSDHL	0.16373	0.15855	-1.03262	No change
OLR1	0.0077	0.00091	-8.44545	Down-regulated***
OSBPL1A	0.04573	0.05616	1.228	No change
OSBPL5	0.01562	0.00077	-20.31702	Down-regulated***
PCR	0.99884	0.75653	-1.3203	No change
PCSK9	0.01624	0.00053	-30.69018	Down-regulated***
PMVK	0.07279	0.05979	-1.21732	No change
PPARD	0.11381	0.12951	1.13787	No change
PRKAG2	0.2307	0.13982	-1.64997	Down-regulated*
RPLP0	16.7169	0.45742	-36.54624	Down-regulated***
SCAP	0.01359	0.00696	-1.95202	Down-regulated*
SCARF1	0.00268	0.00085	-3.13359	Down-regulated**
SNX17	0.14996	0.20833	1.38928	No change
SOAT1	0.07254	0.16556	2.28219	Up-regulated*
SORL1	0.13875	0.20359	1.46739	No change
SREBF1	0.02541	0.03085	1.21439	No change
SREBF2	0.027	0.02399	-1.12553	No change
STAB1	0.01193	0.00053	-22.64322	Down-regulated***
STARD3	0.03829	0.01876	-2.04119	Down-regulated*
TM7SF2	0.57316	0.43579	-1.31522	No change
TRERF1	0.00132	0.00105	-1.25936	No change
VLDLR	0.00851	0.01108	1.30163	No change

Fold-Change (2^(-Delta Delta Ct)) is the normalized gene expression (2^(- Delta Ct)) in TFEB-silenced (shTFEB) cells, divided the normalized gene expression (2^(- Delta Ct)) in wild-type (WT) cells, where Ct is the threshold cycle in qRT-PCR. Fold-change values greater than 1 indicate up-regulation, fold-change values less than 1 indicate down-regulation. The p values are calculated based on a Student’s t-test of the replicate 2^(-Delta Ct) values for each gene. *p<0.05, *p<0.01, ***p<0.001: shTFEB vs WT cells.

Supplemental Table S5. Hematochemical parameters of animals after euthanasia.

	WT				shTFEB			
	**vehicle**	**PT**	**NZ**	**NZ+PT**	**vehicle**	**PT**	**NZ**	**NZ+PT**
**RBC (x 10^6/µl)**	13.23 + 2.01	12.18 + 1.95	13.21 + 1.42	12.18 + 1.69	12.59 + 2.47	11.29 + 2.61	13.48 + 4.52	12.11 + 3.51
**Hb (g/dl)**	13.11 + 1.44	12.45 + 2.31	13.48 + 0.91	12.41 + 1.13	12.63 + 1.94	12.01 + 2.15	12.54 + 1.59	12.04 + 2.51
**WBC (x 10^3/µl)**	13.28 + 3.02	14.37 + 2.39	12.04 + 2.15	13.08 + 1.57	11.07 + 2.07	13.29 + 2.51	12.11 + 1.27	13.45 + 2.45
**PLT (x 10^3/µl)**	873 + 134	745 + 234	916 + 115	8566 + 152	896 + 205	792 + 184	809 + 152	701 + 172
**LDH (U/l)**	9823 + 548	10523 + 627	8912 + 604	7598 + 501	7984 + 412	8216 + 285	7452 + 205	7205 + 236
**AST (U/l)**	156 + 44	135+ 34	143 + 39	144 + 49	134+ 29	146 + 34	152 + 47	162 + 44
**ALT (U/l)**	36 + 14	45 + 11	45 + 19	54 + 23	44 + 18	37 + 11	49 + 15	53 + 11
**AP (U/l)**	114 + 22	127 + 28	129 + 342	113 + 205	128 + 33	108 + 27	119 + 37	109 + 48
**Creatinine (mg/l)**	0.071 + 0.009	0.082 + 0.007	0.069 + 0.011	0.079 + 0.009	0.074 + 0.007	0.079 + 0.010	0.072 + 0.008	0.0692 + 0.011
**CPK (U/l)**	231 + 22	256 + 42	282+ 48	304 + 55	205 + 38	249 + 52	281 + 61	298 + 84

Hematochemical parameters measured immediately after mice euthanasia. RBC: red blood cells; WBC: white blood cells; Hb: hemoglobin (Hb); PLT: platelets; LDH: lactate dehydrogenase; AST: aspartate aminotransferase; ALT: alanine aminotransferase; AP: alkaline phosphatase; CPK: creatine phosphokinase (CPK). Data are means ± SD (n= 5 mice/group treatment).

Supplemental Figures

Supplemental Figure S1

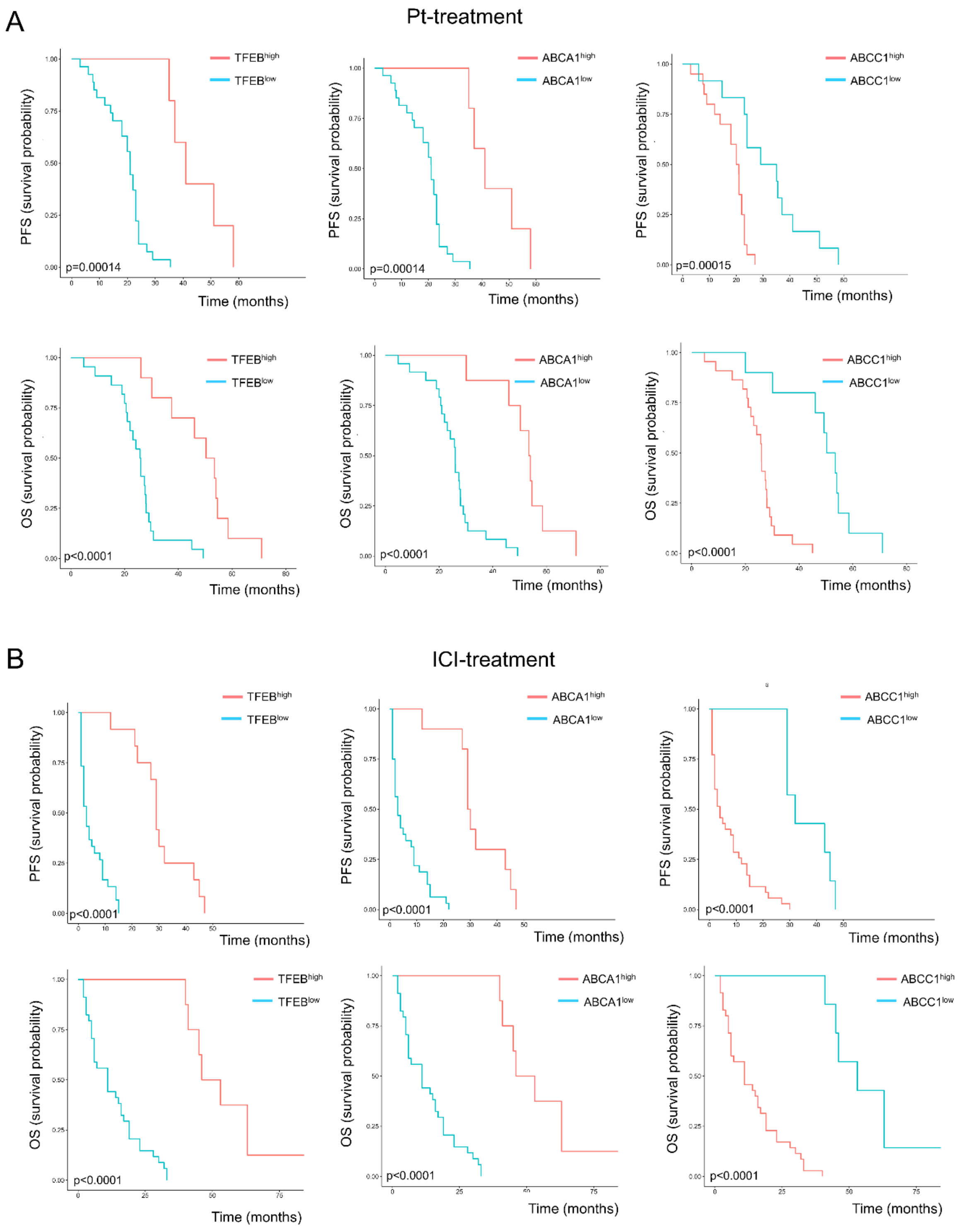

Supplemental Figure 1. **Impact of the expression of TFEB, ABCA1 and ABCC1 on survival of NSCLC patients.** Kaplan Meyer analysis of progression-free survival (PFS) and overall survival (OS) in patients treated with cisplatin/carboplatin (Pt; n=32; panel A) or pembrolizumab as immune checkpoint inhibitor (ICI; n=43; panel B) at the Department of Oncology, University of Torino, Italy. Patients were categorized in "high" and low" according to the median levels of TFEB, ABCA1 and ABCC1.

Supplemental Figure S2

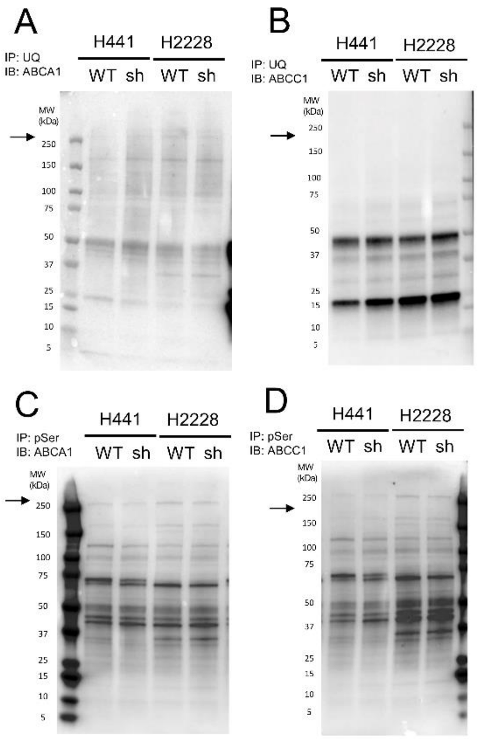

Supplemental Figure S2. TFEB does not affect ubiquitination or phosphorylation of ABC transporters. Ubiquitination (A-B) and phosphorylation (C-D) of ABCA1 (A, C) and ABCC1 (B, D) in wild-type (WT) and TFEB silenced (sh) NCI-H441 and NCI-H2228 cells. Lysates were immunoprecipitated (IP) with an anti-poly-ubiquitination (UQ) or an anti-phosphoserine (pSer) antibody, then immunoblotted (IB) for ABCA1 or ABCC1 (n=3 independent experiments).

Supplemental Figure S3

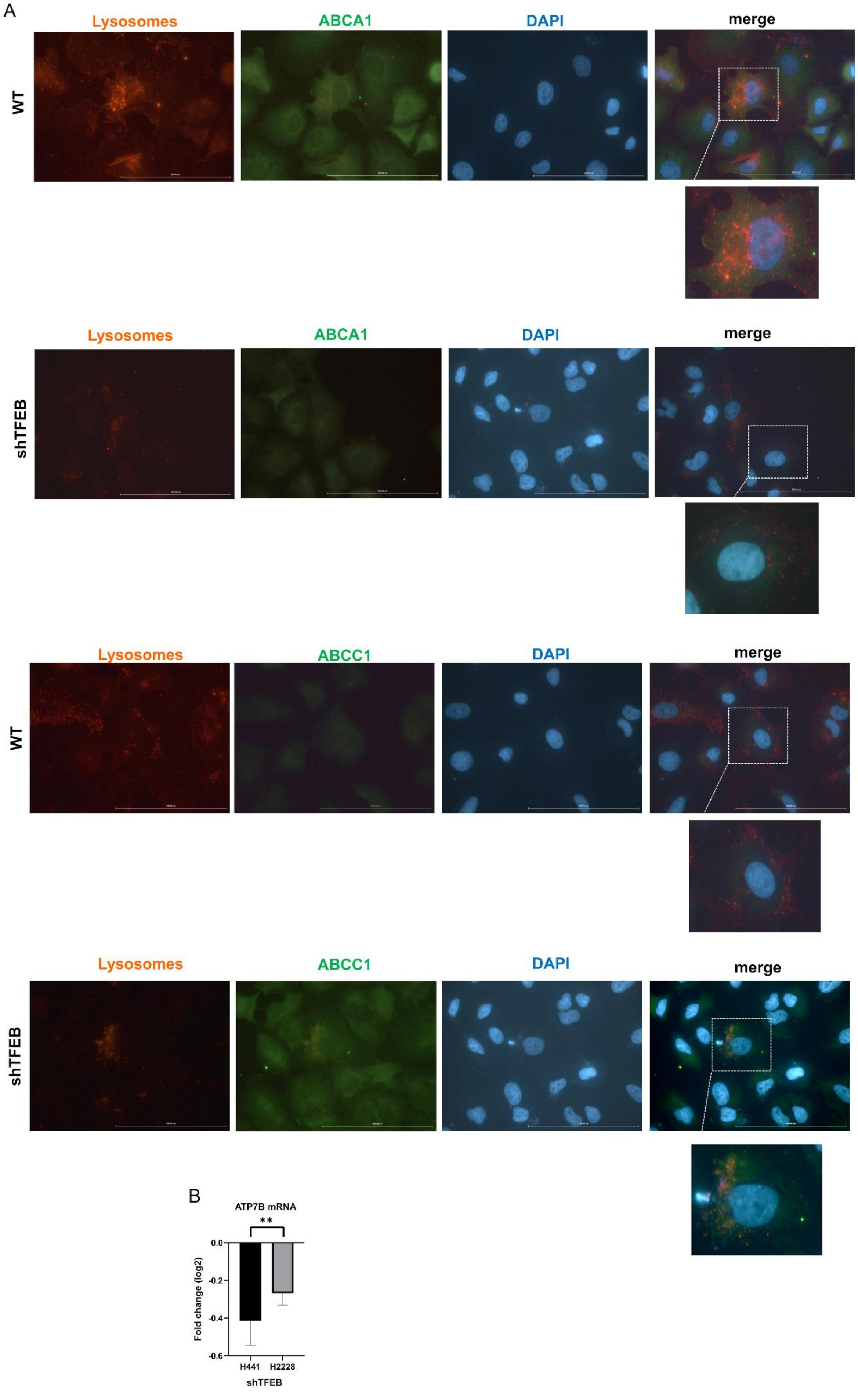

Supplemental Figure S3. Analysis of lysosomes, ABCA1, ABCC1 and ATP7B in non-small cell lung cancer cells

(A). Wild-type (WT) and TFEB-silenced (shTFEB) NCI-H2228 cells were stained with the BioTracker™ 560 Orange Lysosome Dye to label lysosomes plus an anti-ABCA1 or anti-ABCC1 antibody, followed by the DyLight™ 488- or Alexa Fluor488-conjugated secondary antibody. Nuclei were counterstained with DAPI. For each experimental point, a minimum of five microscopic fields were examined. Objective: 63× (1.4 numerical aperture); ocular: 10×. Bar=100 µm. (B). ATP7B mRNA in WT NCI-H441 and NCI-H2228 cells, transduced with the pLKO empty vector, and in cells silenced for TFEB (shTFEB), measured by qRT-PCR in triplicates. Data are means±SD of the mRNA fold change (n=3 independent experiments). **$p<0.01$: shTFEB versus WT cells.

Supplemental Figure S4

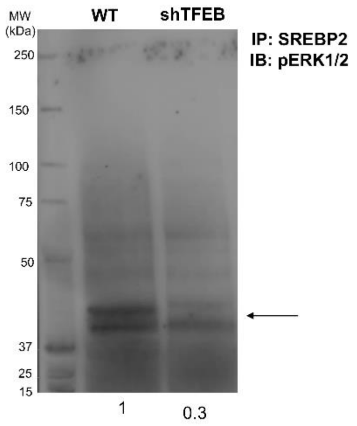

Supplemental Figure S4. Co-immunoprecipitation between SREBP2 and phospho(Thr202/Tyr204)-ERK1/2 in non-small cell lung cancer cells

Lysates from wild-type (WT) and TFEB-silenced (shTFEB) NCI-H2228 cells were immunoprecipitated (IP) for SREBP2, using an antibody recognizing both precursor and cleaved/active SREBP2, then immunoblotted (IB) for phospho(Thr202/Tyr204)-ERK1/2 (n=3 independent experiments). The density of phospho(Thr202/Tyr204)-ERK1/2 in shTFEB cells is indicated below the image, setting the band density of WT cells as 1.

Supplemental Figure S5

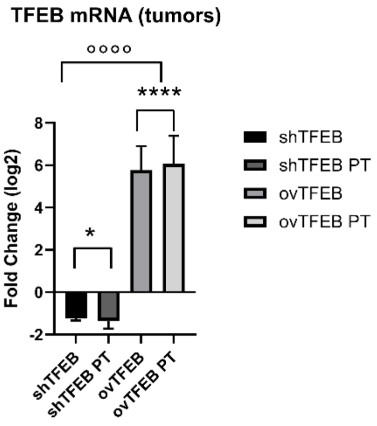

Supplemental Figure S5. Intratumor levels of TFEB after silencing or overexpression. 1×10^6 NCI-H2228 wild-type (WT), TFEB-silenced (shTFEB) cells or shTFEB cells overexpressing TFEBS142A (ovTFEB), mixed with 100 µL Matrigel (Merck), were injected subcutaneously in female NOD SCID-γ (NSG) mice engrafted with human hematopoietic $CD34^+$ cells (Hu-$CD34^+$). When tumors reached the volume of 50 mm3, animals (n=5/group) were randomized and treated for 3 weeks as it follows: 1) control (CTRL) group, treated with 0.1 ml saline solution intravenously (i.v.), once a week; 2) cisplatin (PT) group, treated with 2 mg/kg cisplatin i.v., once a week. Animals were euthanized on day 28 after randomization with zolazepam (0.2 mL/kg) and xylazine (16mg/kg). RNA was extracted from fresh tumors and analyzed for TFEB expression by qRT-PCR, in triplicates (n=5 animals/group of treatment). Data are means±SD. *p<0.05, ***p<0.001: shTFEB/ovTFEB versus WT tumors; °°°p<0.001: ovTFEB vs shTFEB tumors.

Supplemental Figure S6

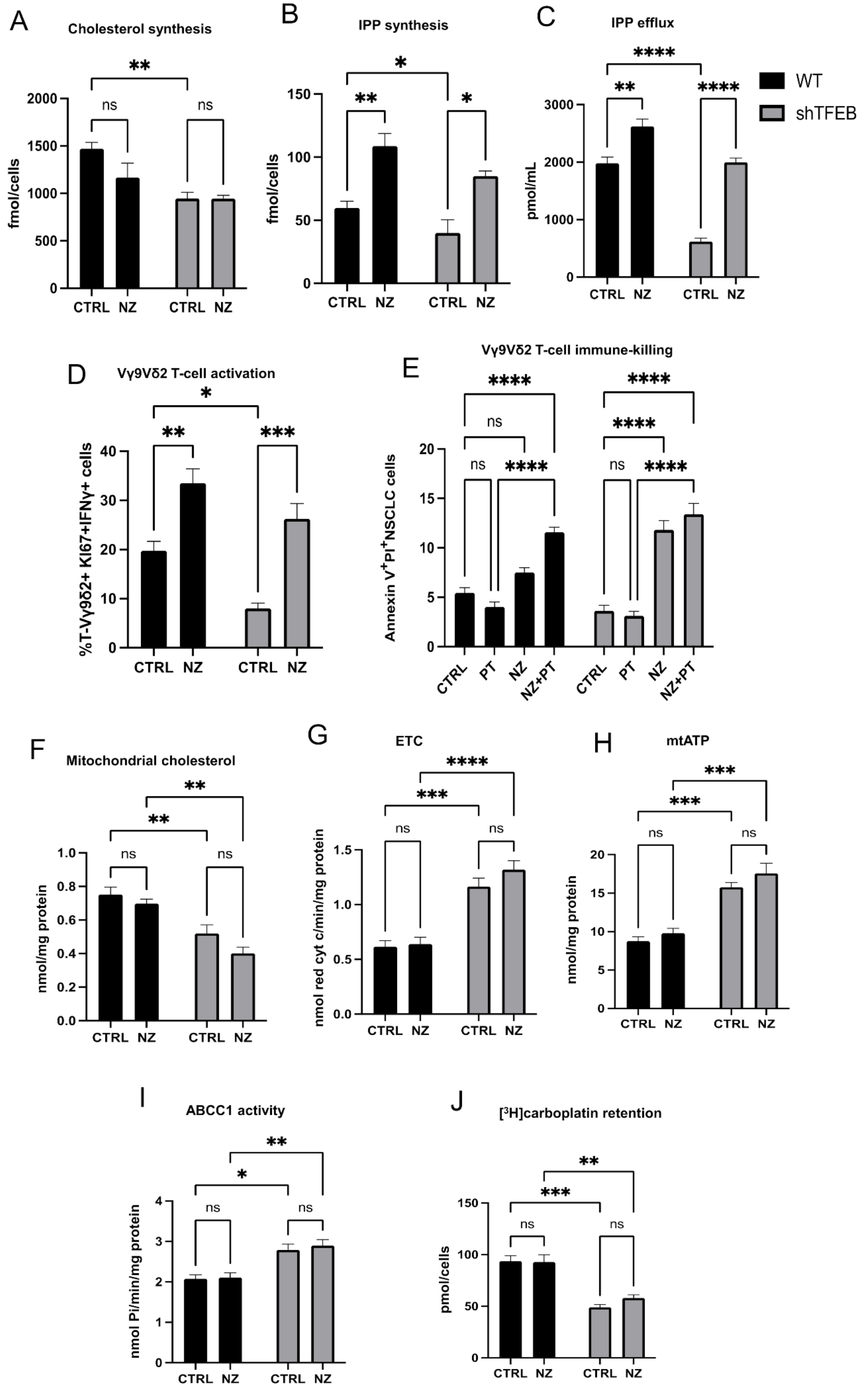

Supplemental Figure S6. Chemo-immuno-sensitizing effects of self-assembled zoledronic acid nanoparticles on TFEB-silenced non-small cell lung cells. Wild-type (WT) or TFEB-silenced (shTFEB) NCI-H2228 cells were incubated 24 h in fresh medium (CTRL) or with 100 nM self-assembled zoledronic acid nanoparticles (NZ). When indicated, 100 µM cisplatin (corresponding to the IC_{25} in WT cells; PT) was added, alone or with NZ. (A-C) Cholesterol synthesis, IPP synthesis and IPP efflux, measured by metabolic radiolabelling in duplicates. Data are means±SD (n=3 independent experiments). *p<0.05, **p<0.01, ****p<0.0001: shTFEB cells versus WT cells. (D-E). Expansion and activation of Vγ9Vδ2 T-lymphocytes, Vγ9Vδ2 T-lymphocyte-mediated NSCLC cell immune-killing, measured by flow cytometry in duplicates. Data are means±SD (n=3 independent experiments). *p<0.05; **p<0.01; ***p<0001, ****p<0.0001: shTFEB versus WT cells. (F-H) Mitochondrial cholesterol and electron transport chain, measured spectrophotometrically in duplicates, mitochondrial (mt) ATP, measured by a chemiluminescence-based assay in duplicates. Data are means±SD (n=3 independent experiments). **p<0.01, ***p<0.001, ****p<0.0001: shTFEB versus WT cells. (I-J). ABCC1 catalytic activity, measured spectrophotometrically, and intracellular retention of [^{14}C]-Carboplatin, measured by cell radiolabelling, in duplicates. Data are means±SD (n=3 independent experiments). *p<0.05, **p<0.01, ***p<0.001: shTFEB versus WT cells.

Supplemental Figure S7

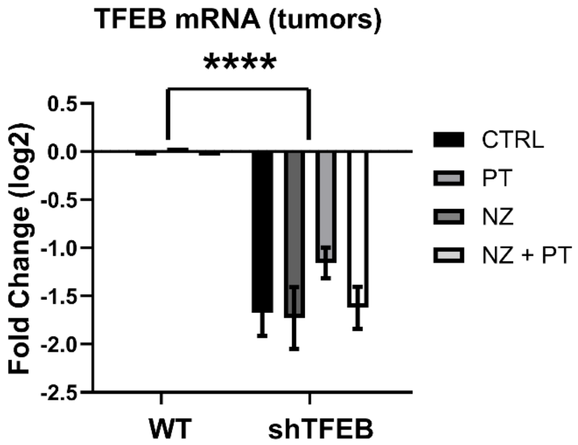

Supplemental Figure S7. TFEB levels in explanted tumors. $1×10^6$ NCI-H2228 wild-type (WT) and TFEB-silenced (shTFEB) cells were injected subcutaneously in Hu-CD34$^+$ mice. When tumors reached the volume of 50 mm^3, animals (n=5/group) were randomized and treated for 3 weeks as it follows: 1) control (CTRL) group, treated with 0.1 ml saline solution intravenously (i.v.), once a week; 2) cisplatin (PT) group, treated with 2 mg/kg cisplatin i.v., once a week; 3) NZ, treated with 1 mg/kg NZ i.v., once a week; 4) NZ+PT group, receiving both drugs i.v., once a week simultaneously. Animals were euthanized on day 28 after randomization with zolazepam (0.2 mL/kg) and xylazine (16mg/kg). RNA was extracted from fresh tumors and analyzed for TFEB expression by RT-PCR, in triplicates (n=5 animals/group of treatment). Data are means±SD. ****p<0.0001: shTFEB versus WT tumors.

Supplementary Figure S8

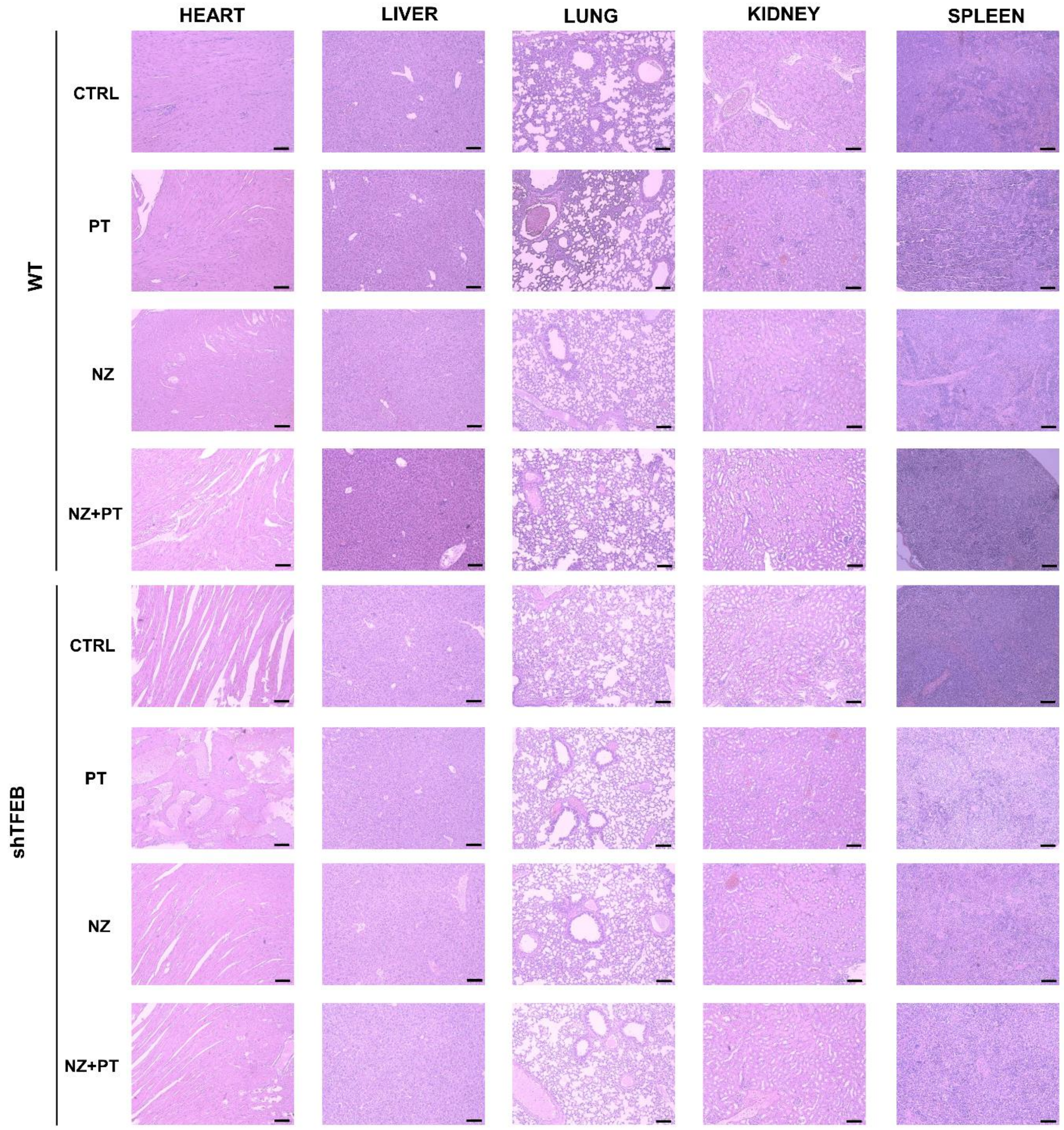

Supplemental Figure S8. Hematoxylin-eosin staining of heart, liver, lung, kidneys, and spleen collected *post-mortem*. The organs were collected from animals of each treatment group immediately after euthanasia, fixed and stained with hematoxylin-eosin. A minimum of 5 field/organ was analyzed in each animal. Objective: 10X; ocular: 10X; Bar =100 μm.

Supplemental Figure S9

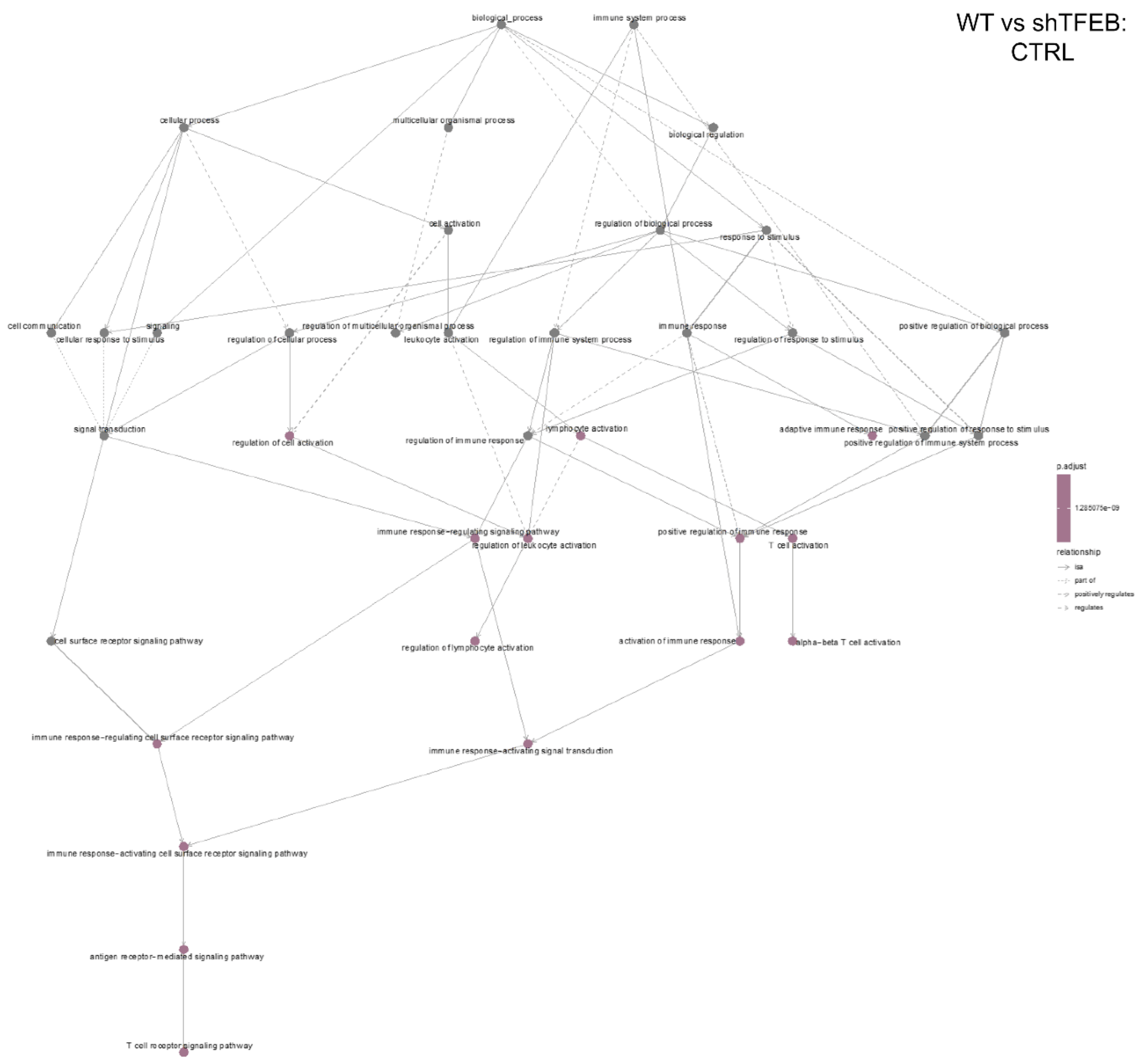

Supplemental Figure S9. Gene network of biological processes differentially up-regulated in wild-type versus TFEB-silenced non-small cell lung cancer tumors. GSEA performed on clustered cancer cells from wild-type (WT) of TFEB-silenced (shTFEB) tumors in animals treated with vehicle (CTRL; n=2 tumors/group). Purple nodes: significantly regulated processes.

Supplemental Figure S10

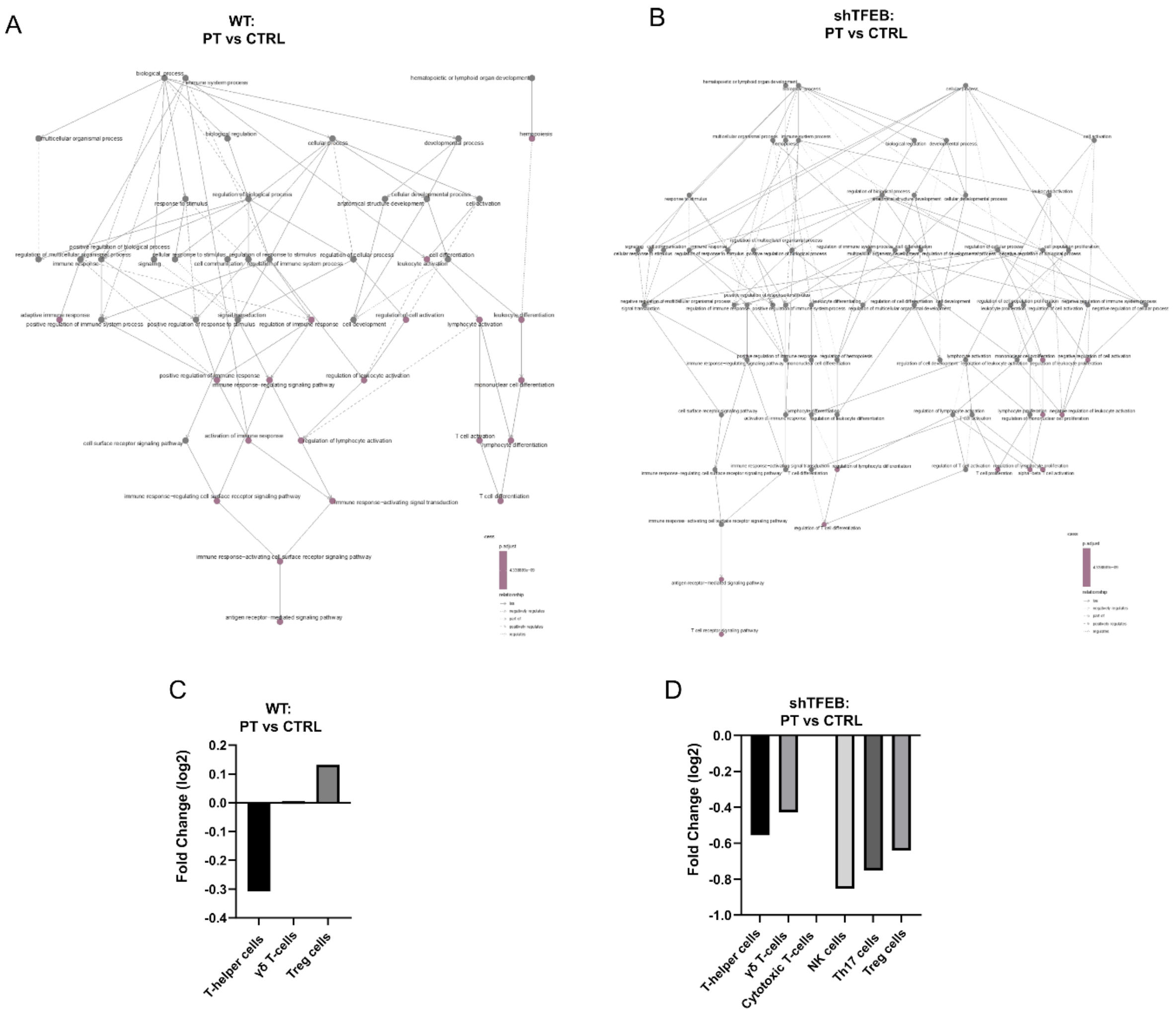

Supplemental Figure S10. Differences in immune-related processes and immune-infiltrating populations in cisplatin-treated non-small cell lung cancers. (A-B). Gene network of biological processes differentially up-regulated in wild-type (WT) versus TFEB silenced (shTFEB) non-small cell lung cancer tumors, based on GSEA performed on clustered cancer cells from animals treated with cisplatin (PT). Purple nodes: significantly regulated processes. (C-D). Quantitative changes, expressed as fold change in a logarithmic scale, of the differentially present immune-infiltrating populations emerged by the comparisons of the following groups: WT PT versus WT CTRL (C), shTFEB PT versus shTFEB CTRL (D; n=2 tumors/group).

Supplemental Figure S11

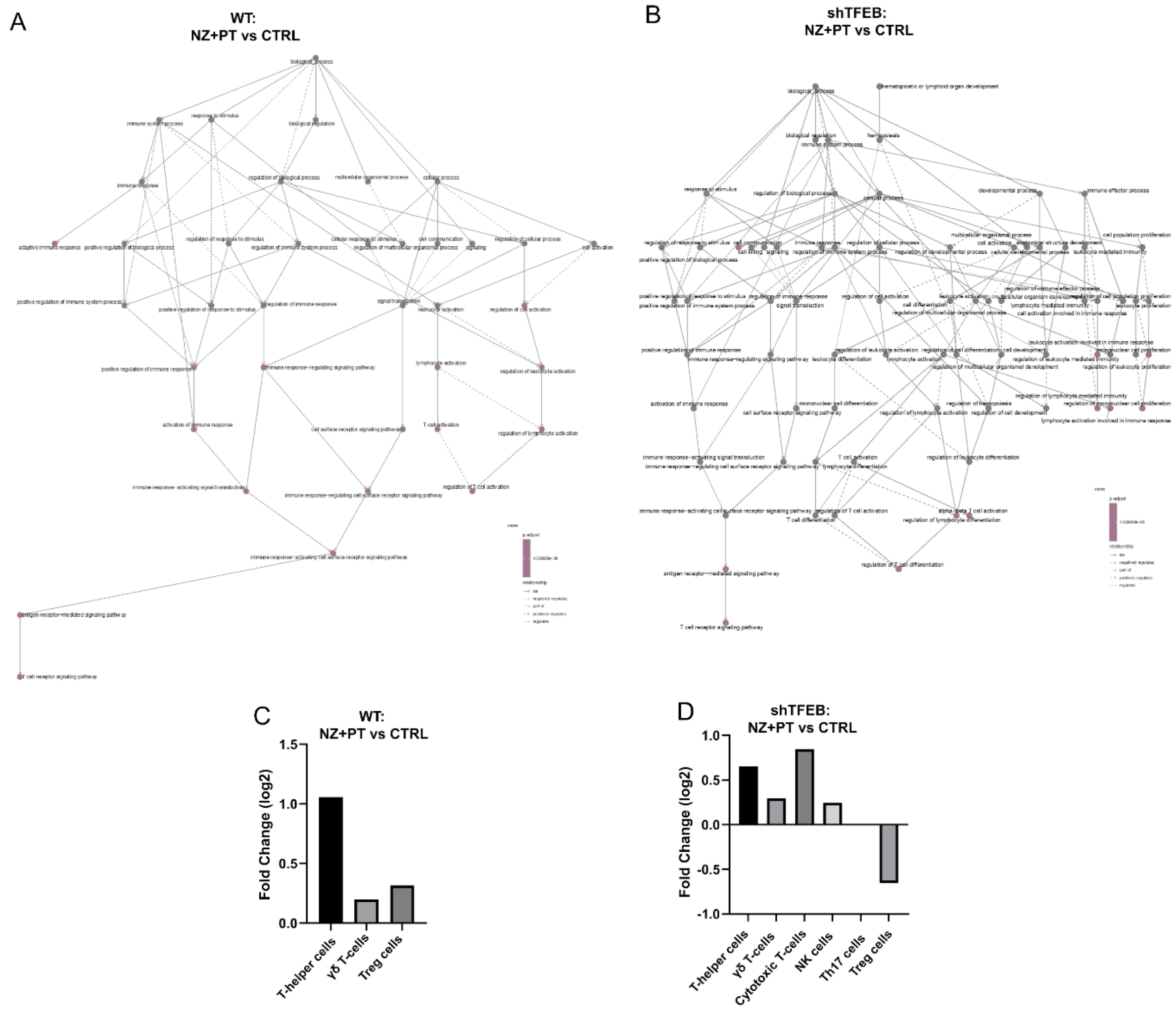

Supplemental Figure S11. Differences in immune-related processes and immune-infiltrating populations in non-small cell lung cancer treated with cisplatin and zoledronic acid. (A-B). Gene network of biological processes differentially regulated in wild-type (WT) versus TFEB silenced (shTFEB) non-small cell lung cancer tumors, based on GSEA performed on clustered cancer cells from animals treated with NZ+PT. Purple nodes: significantly regulated processes. (C-D). Quantitative changes, expressed as fold change in a logarithmic scale, of the differentially present immune-infiltrating populations emerged by the comparisons of the following groups: WT NZ+PT versus WT CTRL (C), shTFEB NZ+PT versus shTFEB CTRL (D; n=2 tumors/group).

Supplemental Figure S12

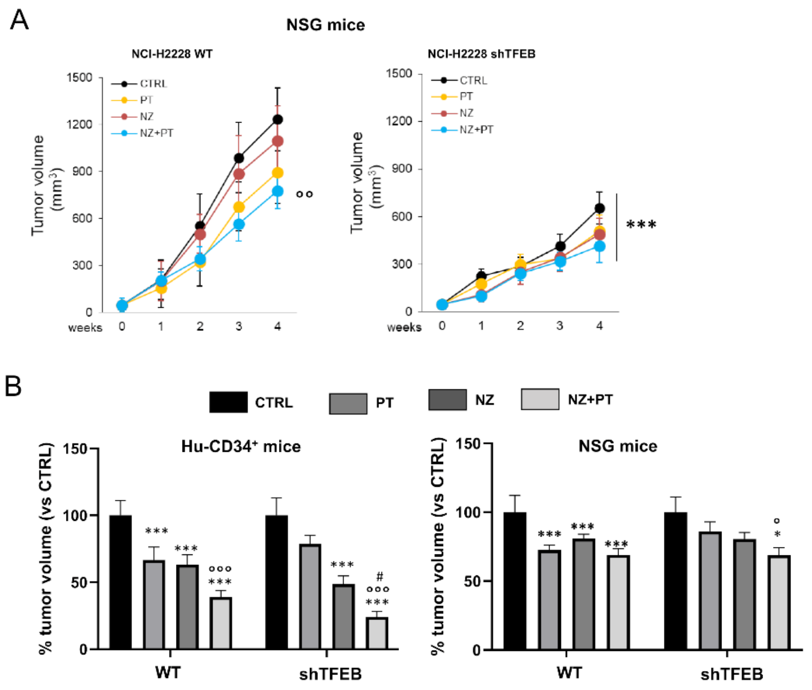

Supplemental Figure S12. Tumor growth in immunocompetent versus immunodeficient mice. (A) 1×10^6 NCI- H2228 wild-type (WT) and TFEB-silenced (shTFEB) cells were injected subcutaneously in female NOD SCID-γ (NSG) mice. When tumors reached the volume of 50 mm^3, animals (n=5/group) were randomized and treated for 3 weeks as it follows: 1) control (CTRL) group, treated with 0.1 ml saline solution intravenously (i.v.), once a week; 2) cisplatin (PT) group, treated with 2 mg/kg cisplatin i.v., once a week; 3) NZ, treated with 1 mg/kg NZ i.v., once a week; 4) NZ+PT group, receiving both drugs i.v., once a week simultaneously. Animals were euthanized on day 28 after randomization with zolazepam (0.2 mL/kg) and xylazine (16mg/kg). Data are means±SD. ***p<0.0001: shTFEB versus WT tumors; °°p<0.01: NZ+PT versus CTRL group. (B) Reduction of volume for tumors implanted in Hu-CD34$^+$ mice (Figure 6A) and NSG mice (panel A). The mean volume in the CTRL group was considered as 100%. The volumes in the other treatment groups were expressed as percentage versus CTRL group. Data are means±SD. *p<0.05, ***p<0.001: treatment groups versus CTRL; °p<0.05, °°°p<0.0001: NZ+PT versus PT; $^{\#}$p<0.05: shTFEB versus WT tumors.
